# De Novo Genesis of Enhancers in Vertebrates

**DOI:** 10.1371/journal.pbio.1001188

**Published:** 2011-11-01

**Authors:** Michael P. Eichenlaub, Laurence Ettwiller

**Affiliations:** Centre for Organismal Studies, University of Heidelberg, Heidelberg, Germany; Duke University, United States of America

## Abstract

Evolutionary innovation relies partially on changes in gene regulation. While a growing body of evidence demonstrates that such innovation is generated by functional changes or translocation of regulatory elements via mobile genetic elements, the de novo generation of enhancers from non-regulatory/non-mobile sequences has, to our knowledge, not previously been demonstrated. Here we show evidence for the de novo genesis of enhancers in vertebrates. For this, we took advantage of the massive gene loss following the last whole genome duplication in teleosts to systematically identify regions that have lost their coding capacity but retain sequence conservation with mammals. We found that these regions show enhancer activity while the orthologous coding regions have no regulatory activity. These results demonstrate that these enhancers have been de novo generated in fish. By revealing that minor changes in non-regulatory sequences are sufficient to generate new enhancers, our study highlights an important playground for creating new regulatory variability and evolutionary innovation.

## Abstract 2

Whole genome duplication in teleost fish reveals that a few changes in non-regulatory genomic sequences are a source for generating new enhancers.

## Author Summary

The genome of each living organism contains thousands of genes, and the precise control of the timing and location of expression of these genes is key for normal development and homeostasis of each individual. Despite the oftentimes high genetic similarity between organisms, the source of phenotypic differences, for example between human and mouse, is thought to originate mainly from changes in how and when genes are expressed. This is partially determined by enhancers, that contribute to the control of gene expression. For decades, duplication of existing genomic enhancers, mobile elements, and changes in the sequence of existing enhancers were believed to be the major ways of increasing the number and modifying the activity of enhancers. In this study, we show that enhancers don't have to be derived from pre-existing ones but can also appear de novo in regions of the genome that were previously not regulating gene expression. We analyzed teleost fish genomes and found three regions for which a limited number of changes in the DNA sequence was sufficient to generate new enhancers. We predict that such a process is frequent in vertebrate genomes, making de novo generation of enhancers an important mechanism for creating variation in gene expression.

## Introduction

The question of the evolutionary origin and modification of enhancer elements is central for understanding the dynamics of gene expression [1]–[3]. A growing body of evidence points out that new enhancers evolve from existing ones via duplication. According to the classic model of evolution by duplication as put forward by Ohno [4], the duplicated copies are used as starting material for variation in the binding site composition, which modifies the respective enhancer's activity [5]–[10]. Mobile genetic elements have also been shown to have regulatory activity [11],[12] or bear transcription factor binding sites (TFBSs) [13], and thus, their translocation can be associated with changes in gene expression.

While the modification/translocation of those pre-existing elements has been shown to play an important functional role, they may only contribute to a fraction of the regulatory innovation. Indeed, recent findings using large-scale comparative analysis of regulatory features have shown that single binding sites can vary extensively between closely related species [14] or even between individuals of the same species [15]. Further supporting the flexibility of regulatory elements, tissue-specific enhancers such as heart enhancers have been shown to be poorly conserved [16] and examples of lineage/specie-specific enhancers have been described [17],[18]. Recently it has been reported that the genomic positions of tissue-specific enhancers of the *yellow* gene differ between *Drosophila* species [19].

Taken together, these results are suggesting that complete autonomous enhancer elements containing all the necessary binding sites in the correct arrangement can be lineage specific. Nevertheless it is currently unclear whether these apparent lineage-specific enhancers appear de novo or are derived from pre-existing enhancers whose sequences have diverged too much to be identifiable. In order to show the de novo nature of these lineage-specific enhancers, a strategy to identify the orthologous regions and test them for enhancer activity is needed.

**Figure 1:**
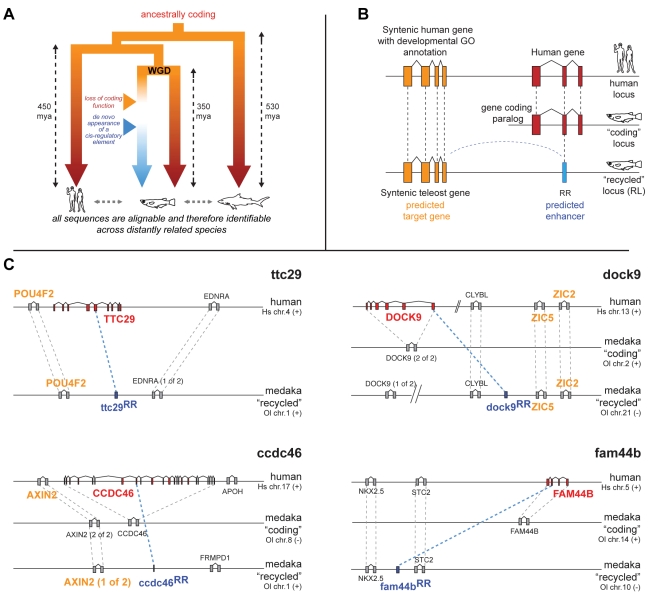
Using an evolutionary trap to identify de novo enhancer sequences. (A) After Whole Genome Duplication (WGD) in teleost fish, one copy of an ancestrally coding sequence lost its coding function and acquired a regulatory function instead (blue branch). The sequence is retained in the fish genome because of the selection acting on the new function while the orthologous sequences in mammalian and shark are retained because of the coding function (red branches). If the gain of the regulatory activity happened sufficiently fast after the loss of the coding function, all sequences (human, fish, and shark) can be detected using standard alignment algorithms. Thus, the de novo nature of the regulatory function in fish is addressable. The evolutionary time since the divergence from the last common ancestor is taken from [35] and [63]. (B) Schematic example of an evolutionary scenario leading to the appearance of de novo enhancers: The recycled locus (RL) in fish has lost the gene (red), but one region (blue) in the RL is still alignable to one human exon. This region we refer to as a “Recycled Region” (RR) and is a good candidate for having a de novo acquired regulatory function controlling the expression of the neighbouring gene (orange). The approach used to identify such a scenario is described in Figure S1, Materials and Methods and Computational Pipeline. (C) Schematic overview of human loci and both duplicated loci in medaka for all candidate RRs: As in (B), the human gene corresponding to the RR is shown in red, the medaka RR is shown in blue. The putative target gene(s) of the RR is in orange. For ttc29^RR^ the homologous coding sequence was not found in medaka. The presence of two intact *dock9* genes in medaka is likely the result of an additional duplication unrelated to the WGD in fish. Only genes flanking the RR and their orthologs are shown. For clarity, all genes (except for the red genes) are shown as two exon genes, even though they may contain more exons. Gene names written above the schematic representation indicate the location on the “plus” strand, names written below indicate location of the gene on the “minus” strand. The distances between genes do not reflect the actual genomic distances.

In this report we identify de novo enhancers by searching for special cases that we refer to as “Recycled Regions” (RRs). An RR is a region with enhancer function in one lineage that remains identifiable in another lineage due to sequence constraints imposed by a different kind of function. These scenarios are likely to be very rare in stable genomes. Thus, we took advantage of the most recent Whole Genome Duplication (WGD) in teleosts [20] followed by a massive loss of the duplicated coding genes. It is estimated that 75% of the duplicated genes lost one copy [20]. Initially, while one of the duplicated copies remained a coding gene, the other copy lost its coding function and accumulated nucleotide changes. In rare cases, the sequence from the non-coding copy became constrained if a regulatory function arose de novo. Those regulatory sequences are alignable to their coding orthologs if the selection for the new function took place soon enough. Hence we used the ancestral coding function as an evolutionary trap to identify orthologous sequences of the enhancer across lineages (mammalian, cartilaginous fish, and teleost) and assessed whether these enhancers are generated de novo in the teleost lineage (Figure 1A).

## Results

### Identification of the Recycled Regions

We developed an algorithm to systematically search for the RRs in teleost fish genomes that satisfy the corresponding criteria (Figure 1B): (1) are located in the locus corresponding to the lost copy of a duplicated gene; (2) despite no evidence for the coding function, are conserved with part of the human coding ortholog; and (3) as experimental validation is performed during embryogenesis, we selected those RRs flanked by at least one gene annotated to be involved in development (Figure S1 and Materials and Methods, Computational Pipeline). The algorithm was first run on the stickleback (*Gasterosteus aculeatus*) genome because of the high quality of the gene annotation and assembly, and later the results were transferred to the *Oryzias latipes* (medaka) genome. Our analysis identified four RRs (Figure 1C, Table S1, and Table S2) as putative de novo regulatory regions satisfying the above criteria. Those RRs are conserved across teleosts including *Danio rerio* (zebrafish), suggesting that they appear after the WGD but before the Cypriniformes-Euteleostei split.

### The Recycled Regions Show Enhancer Activity

**Figure 2:**
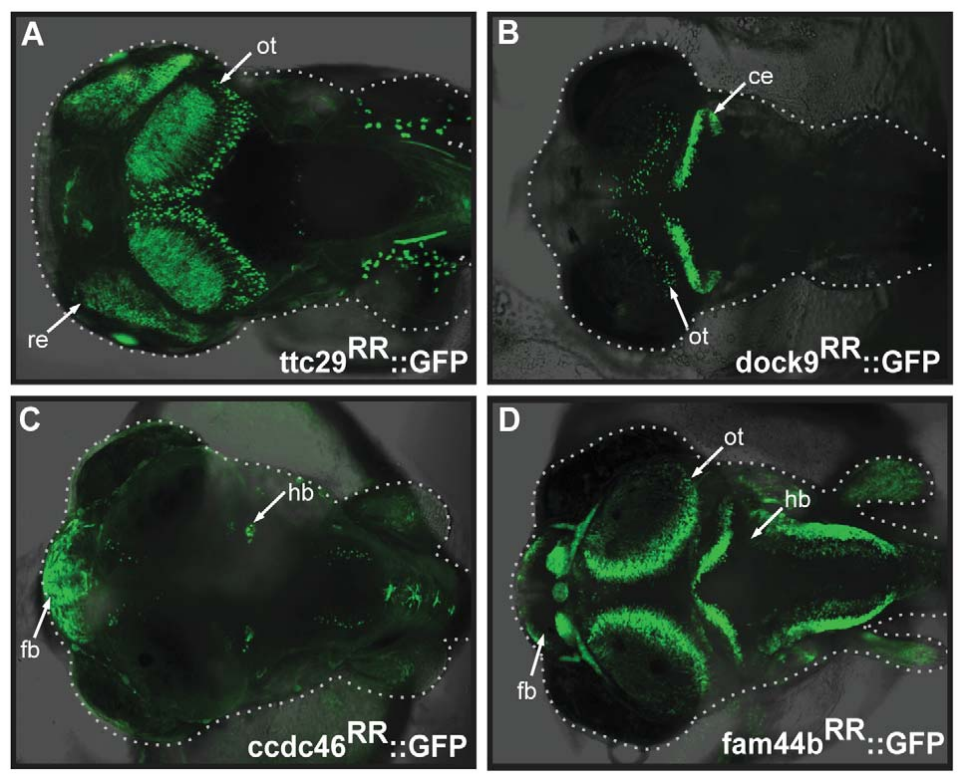
The Recycled Regions (RRs) show enhancer activity in medaka. Expression of the reporter gene GFP under the control of the RRs (± flanking 200 bp) in stable transgenic embryos. Confocal images of medaka stage 32 embryos (dorsal view, anterior to the left). (A) ttc29^RR^::GFP construct: The reporter gene can be detected in the retina (re) and in the optic tectum (ot) when driven by the ttc29^RR^. The lens expression is attributed to the activity of the hsp70 minimal promoter (see Methods). (B) dock9^RR^::GFP construct: The reporter gene can be detected in the cerebellum (ce) and cells in the optic tectum (ot). (C): ccdc46^RR^::GFP construct: The reporter gene can be detected in fore- and hindbrain (fb/hb). (D) fam44b^RR^::GFP construct: The reporter gene can be detected in multiple structures of the brain, including forebrain (fb), optic tectum (ot), and hindbrain (hb).

We investigated the enhancer activity of the four medaka RRs (Figure 1C and Table S1) using an in vivo reporter assay in medaka that we previously developed [21]. We cloned the four RRs extended with a maximum of 200 bp flanking sequences upstream of an hsp70 minimal promoter and a reporter gene (*gfp*). The basal expression of the hsp70 minimal promoter in the lens [22] was used as injection control. We found that all four regions tested drive reporter gene expression in specific structures in the medaka embryo (Figure 2A–D). The assay is highly reproducible, resulting in a consistent expression pattern in a large fraction of embryos (Table S3). The onset of reporter gene expression depends on the nature of the RR and varies from developmental stage 20 (fam44b^RR^) to stage 32 (dock9^RR^) and is in all cases maintained in juvenile (Figure S2) and adult fish (unpublished data). Moreover, the specific expression pattern observed in injected embryos (Table S3) is retained in stable lines. In line with our hypothesis, these results show enhancer activity for all four RR reporter constructs. We further addressed the contribution of the four RRs to the observed enhancer activity by deleting the orthologous regions corresponding to the exon, leaving only the flanking regions from the reporter constructs (Figure S3A–D). In two cases, the deletion constructs completely abolished reporter gene expression (Figure S3E–F). For ccdc46^RR^, the deletion altered and massively reduced the reporter gene expression to a few cells in the hindbrain (Figure S3G). Only for fam44b^RR^ did the deletion construct not abolish the original enhancer activity of the full construct (Figure S3H) and therefore fam44b^RR^ was excluded from further analysis. These results demonstrate that three out of four RRs are necessary for enhancer activity.

### Recycled Regions Recapitulate Part of the Flanking Gene Expression Patterns

**Figure 3:**
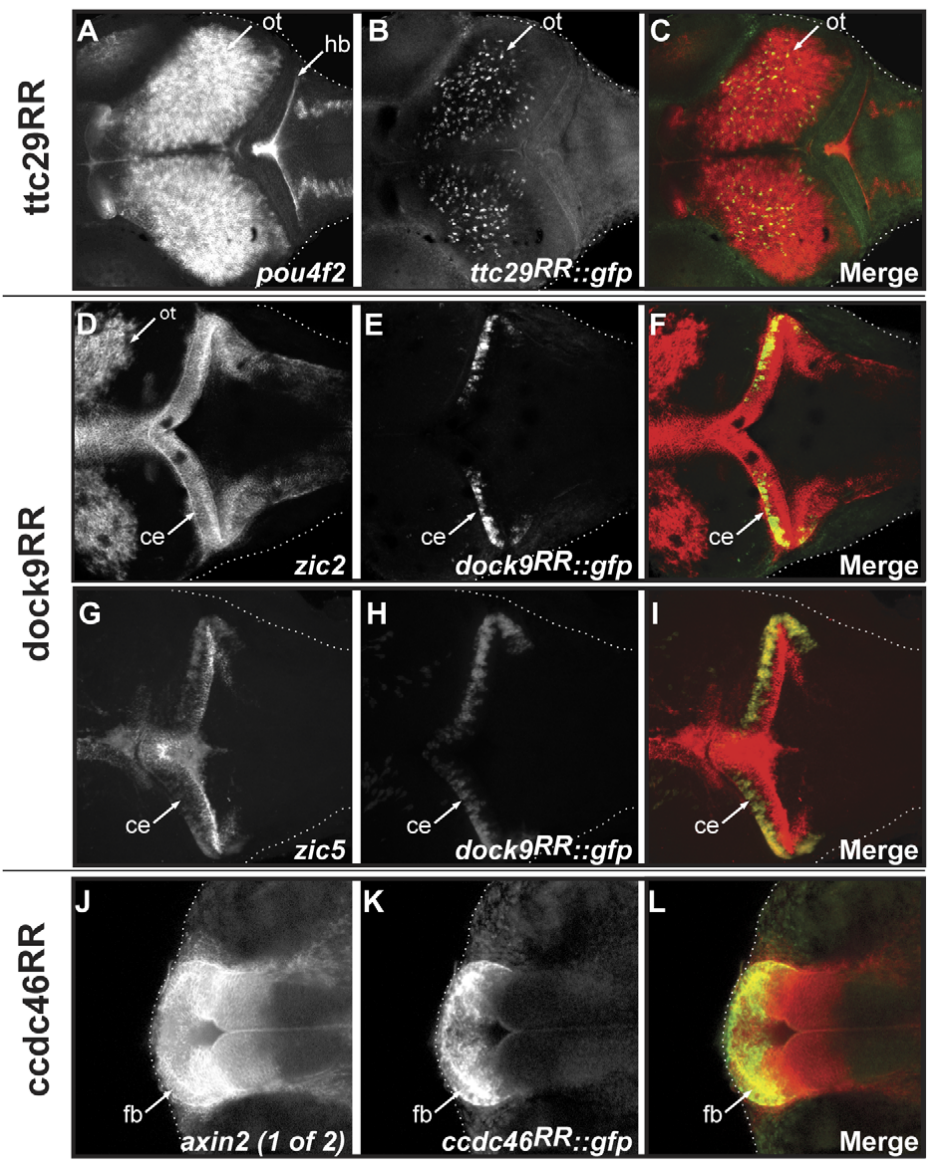
Enhancer activity of the RRs recapitulates key aspects of the neighbouring gene expression pattern. Double-fluorescent whole-mount in situ hybridisation of candidates. The mRNA of the putative target gene was visualised using Fast Red staining (A, D, G, J shown in red in the merged images). GFP mRNA driven by the RRs was detected using TSA-fluorescein (B, E, H, K shown in green in the merged images). Confocal images of medaka stage 32 embryos (dorsal view, anterior to the left). (A–C) Expression of *pou4f2* mRNA is detected in the optic tectum (ot) and the hindbrain (hb) (A) while ttc29^RR^ drives GFP mRNA expression in a subset of cells in the optic tectum (ot) (B). Both expression patterns overlap in the optic tectum (ot) (C). (D–F) Expression of *zic2* mRNA is detected in the optic tectum (ot) and the cerebellum (ce) (D) while dock9^RR^ drives GFP mRNA expression specifically in the cerebellum (ce) (E). Both expression patterns overlap in the cerebellum (ce) (F). (G–I) Expression of *zic5* mRNA is detected in the cerebellum (ce) (G) while dock9^RR^ drives GFP mRNA expression in the cerebellum (ce) (H). Both expression patterns overlap in the cerebellum (ce) (I). (J–L) Expression of *axin2 (1 of 2)* mRNA is detected in the forebrain (fb) (J), as well as for the GFP expression under the control of ccdc46^RR^ (K). Both expression patterns overlap in the anterior part of the forebrain (L).

We next investigated whether the enhancer activity of the remaining three RRs recapitulates aspects of the expression pattern of flanking genes. For this, we analysed the in situ expression pattern of those genes. We found that in all cases RR-driven reporter gene expression temporally and spatially resembles the expression of at least one of the respective flanking genes (Figure S4). To further confirm this, we performed double fluorescent whole mount in situ hybridisation on stable transgenic lines by combining probes for the reporter and the flanking genes. In all cases, we identified at least one flanking gene that recapitulates key aspects of the expression pattern of the RR-driven reporter gene (Figure 3). In particular, both ttc29^RR^-driven GFP (Figure 3B) and the flanking gene *pou4f2* (Figure 3A) are expressed in the optic tectum and retina (Figure 3C). dock9^RR^ shows very specific enhancer activity in the cerebellum (Figure 3E,H) as do the neighbouring genes *zic5* and *zic2* (Figure 3D,G), which exhibit an expression pattern that includes the cerebellum (Figure 3F,I). Finally, ccdc46^RR^ shows activity in the forebrain (Figure 3K), recapitulating part of the expression pattern of its flanking gene *axin2 (1 of 2)* (Figure 3J,L). All putative target genes have been reported to play important roles in developmental processes: Zic2 and 5 are zinc finger proteins of the cerebellum, and mutations in the *zic2* gene have been reported to cause holoprosencephaly [23]. Axin2, an Axin-related protein, has been shown to play an important role in the regulation of β-catenin stability in the Wnt signalling pathway [24], and Pou4f2, better known as Brn3b, is a member of the POU-domain family of transcription factors and is a key regulator for axon outgrowth and pathfinding in projection neurons [25]. Our results demonstrate that the RRs exhibit enhancer activity that recapitulates multiple aspects of the expression of neighbouring genes. Our results further suggest that the identified RRs contribute to the transcriptional regulation of genes that are key players in embryonic development.

Two possible evolutionary scenarios may account for our results obtained so far: (1) the ancestral function was both regulatory and coding or (2) the ancestral vertebrate sequence was coding but the teleosts have lost that function in one of the duplicated copies and acquired regulatory function instead (which supports the de novo enhancer hypothesis). For the former scenario, dual functions on the same region have been hypothesised [26] and shown for several cases [27]–[32] while the latter scenario has not been shown so far. To shed light on the ancestral state of the RRs, we investigated the RRs in lineages that diverged prior to the last WGD in teleosts.

### Orthologous Regions in Non-Teleost Lineages Show No Enhancer Activity

In species that have diverged prior to the teleost-tetrapod split (e.g., elephant shark (*Callorhinchus milii*) or ciona (*Ciona savignyi*)) the sequences corresponding to the three RRs showed an open reading frame (ORF) spanning the coding exon that is in frame with the human ORF (Figure S5). For both *TTC29* and *CCDC46* we also found EST evidence in the ciona lineage (Table S2). These results show that the RRs ancestral sequences were very likely to have been coding at the split of the teleost-tetrapod lineages.

We next investigated the evolutionary dynamics of these regions by analysing the similarity between the human coding exon and the orthologous regions in various lineages at both the amino-acid (AA) and nucleotide level. We found that the percentage identity at the nucleotide level is higher for the fish RRs, while the similarity at the AA level is higher for all other lineages, including the fish coding paralog (Figure S6). Consistent with the alignment similarities, the ratio of non-synonymous compared to synonymous base pair changes (Ka/Ks) [33] is increased for the RRs compared to the coding homologs (see Materials and Methods and Figure S6). In accordance with the results obtained so far, these data further support the hypothesis that (1) the RRs were ancestrally coding and (2) the fish RRs are under a selection acting at the nucleotide rather than at the AA level. These data suggest that the RRs were ancestrally not regulatory since the Ka/Ks ratio between human and shark or ciona would favour a selection acting at the AA level only.

**Figure 4:**
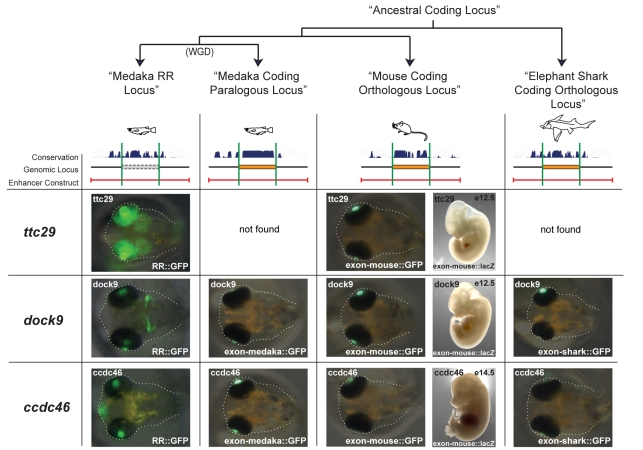
The coding homologs of the RRs show no enhancer activity. The coding homologs of the RRs in medaka (column 2), mouse (column 3), and elephant shark (column 4) show no enhancer activity. For clarity, we included the result of enhancer activity of the RR regions (column 1). For ttc29^RR^ no homologous coding sequences were found in medaka and shark. While only one stage is represented, the monitoring of the reporter gene expression is performed throughout the embryonic development (except for the mouse transgenic assay where the specific stage is annotated on the figure). For dock9^exon-medaka^, both exons from both paralogs were tested (Table S3). Branch lengths and loci are schematic.

To test the nature (regulatory or non-regulatory) of the ancestral state at the tetrapod-teleost split, we further explored the enhancer activity of the exons homologous to the RRs in two independent lineages (mouse and elephant shark) as well as the coding paralog in fish (Figure 4).

In none of the cases tested was an enhancer activity detectable (Figure 4 and Table S3). As the exon orthologous to the RRs was tested in the Medaka embryo, the absence of activity could be due to trans-regulatory changes [34]. To rule out this hypothesis, the mouse exons orthologous to the RRs were tested directly in mouse. Again, in none of the cases tested was an enhancer activity detectable (Figure 4 and Material and Methods), confirming that the mouse exons orthologous to the RRs have no enhancer activity (at the time point assayed).

The results obtained so far provide convincing evidence that the enhancer function in teleosts was de novo acquired in this lineage. As most of the de novo genesis of enhancers is expected to occur in “neutrally” evolving sequences, these cases of de novo enhancers deriving from cooption may constitute a very small subset of all possible de novo enhancers.

We roughly estimate at several thousands the number of de novo enhancers under positive selection since the tetrapod-teleost split (450 mya [35], see Text S1 and Figure S7 for a more detailed analysis of the estimation of the number of de novo enhancers). Considering that those de novo elements under purifying selection may constitute only a tiny fraction of all possible regulatory elements generated, the rate of genesis of new enhancers (regardless of their evolutionary fate) may be very high in vertebrate genomes. While this estimation of the number of de novo enhancers is only tentative and based on a number of assumptions (see Text S1), a more accurate prediction of the de novo enhancers across various phylogenetic branches of vertebrates will require further studies. Nonetheless, these results highlight the importance of the genesis of enhancers and provide one possible explanation amongst others of the widespread observation that a large fraction of TFBSs appears non-conserved [36]. Nonetheless, those TFBSs forming de novo enhancers may represent only a fraction of all the apparent lineage-specific binding sites found by genome-wide chromatin immunoprecipitation experiments.

In an attempt to predict what the possible TFBS involved in the generation of the de novo enhancers are, we further investigated at the sequence level the difference in terms of putative TFBSs between the RRs and the exons (Materials and Methods). We found from five to seven binding sites in the medaka RRs that are specific to teleosts and are not present in other vertebrate species nor in the predicted ancestral reconstruction (Figure S8). Interestingly, dock9^RR^ in medaka (with enhancer activity in the cerebellum) has a new binding site for Pax2, a transcription factor known to be involved in cerebellum development [37].

### Function of the De Novo Enhancers in Gene Regulation

These de novo enhancers may either confer additional domains of expression to their target genes or rather act as redundant enhancers. To tackle the functional consequences of the de novo enhancers, we took advantage of a conserved block flanking the ccdc46^RR^ homologous exon previously shown to be bound by p300 in mouse forebrain (Figure S9, orange bar, upper panel) [38]. We tested the mouse extended region containing both the p300 pulldown region and the extended exonic sequence (Figure S9, light green bar, upper panel) and detected enhancer activity in the medaka forebrain (Figure S9A). This activity was not altered when deleting the exonic sequence (Figure S9, blue bar, upper panel and Figure S9B), demonstrating that the exon itself is not required for enhancer function (see also Figure 4). Similarly, the shark and medaka sequences (Figure S9, orange bar, lower panel) orthologous to the mouse p300-bound enhancer also show forebrain activity (Figure S9C–D). These results demonstrate that the p300-bound enhancer element is an ancestral feature and suggest that the nearby ccdc46^RR^ de novo enhancer in fish has complementary function to reinforce the forebrain expression rather than creating a new expression domain. Similarly dock9^RR^ is active in the medaka cerebellum, while the mouse *zic*2 and 5 genes are also expressed in this structure [39].

While those de novo enhancers may still quantitatively modify the transcript level within the cell or activate transcription in related cell types within the same domains, these results favour the hypothesis of redundant enhancer. This hypothesis is supported by the recent finding that redundant enhancers confer phenotypic robustness [40],[41] and thus are likely to be selected for.

Similar to TFBS turnover by the de novo emergence of new binding sites [42], complete enhancers may also be turned over, leading to the disappearance of the ancestral element.

## Discussion

It has long been thought that new functions emerge primarily by duplication and/or modification of existing functional elements [43]. On the gene level, this view has begun to change with the recent publication of several studies reporting the de novo origin of genes in yeast [44], drosophila [45], and human [46]. In this study we show that not only genes but also enhancers can be de novo generated.

De novo genesis of enhancers raises the question of how evolution can produce such complex functional elements. Indeed, enhancers were generally believed to have a stringent regulatory code, and thus the odds for generating a de novo enhancer were believed to be low. Recent studies have already started challenging that view by pointing either to the flexibility of this code [18],[47] or the rapid turnover of binding sites [14],[15],[42]. It is possible that the appearance of new binding sites can not only modify pre-existing enhancer but also lead to the creation of completely new autonomous enhancers.

This work further shows the relative “facility” of conferring regulatory activities to non-regulatory sequences. Consequently, the birth of regulatory elements is a highly dynamic property of vertebrate genomes and should also be considered as an evolutionary toolkit for innovation. The results of this study have significant implications, notably in the gene regulation and medical genetic fields by pointing out that genomic variation could lead to the generation of enhancers in regions with no apparent regulatory function. As such variation may also lead to altered gene expression, more attention should be devoted to variation in so-called “neutral” DNA.

## Materials and Methods

### Computational Pipeline

#### Summary of the computational pipeline

In order to find RRs we undertook a conservative analysis of the stickleback non-coding genomic sequences mapping to the human exome. For this, a total of 282,599 human annotated exons were mapped to the stickleback genome using BLASTZ. BLASTZ is a sensitive alignment tool suited for non-coding genomic sequences. In order to retain only the stickleback non-coding regions, hits matching even partially an annotated exon in stickleback were removed. To identify putative RRs we took advantage of the last WGD in teleosts followed by the massive loss of the duplicated genes. Only hits in the syntenic loci between human and stickleback were further processed. From the WGD, two such syntenic loci per human locus can be found in fish (one locus contains the functional protein, while the other locus has lost the gene). Thus we restrict the search to only hits containing stop codon(s) disrupting the ORF and found in the locus of the lost gene. Such hits are good candidates for having acquired a de novo enhancer function controlling nearby genes. As experimental validation is performed during embryogenesis, we further selected those hits flanked by at least one gene annotated to be involved in development (Figure 1B and Figure S1).

We identified four BLASTZ hits on the stickleback genome as putative RR candidates and transferred the hits to the medaka genome (Figure 1C and Table S1) for experimental validation.

#### Human exons

The repeat-masked DNA sequences of a total of 282,599 human annotated exons (length >19 bp for BLASTZ) were retrieved from EnsEMBL v. 49 [48].

#### Alignment with stickleback (*Gasterosteus aculeatus*)

DNA sequences corresponding to the human (*Homo sapiens*) exons were matched to the repeat-masked stickleback genome (EnsEMBL v. 49) using BLASTZ (default parameters, score above 2,900) [49]. A total of 145,095 human exons (51%) have at least one BLASTZ hit on the stickleback genome. This number corresponds to 24,214 human genes. The average BLASTZ score is 5,220. The average number of hits on the stickleback genome is 7.3 hits per conserved exon. For each exon, hits on the stickleback genome within 1 kb from each other are considered to be part of the same regulatory unit and were therefore fused. To deplete the dataset from un-annotated genes or exons, only exons from human genes with at least one annotated ortholog in stickleback were further considered. Any hits within 2 Mb of the stickleback ortholog(s) locus were removed. Alignments matching even partially an annotated exon or EST in stickleback or any other sequenced teleosts (EnsEMBL gene annotation) were also removed.

#### Synteny assessment

All the neighbouring developmental genes (see section below) within a 300 kb window upstream and downstream of the human exon were retrieved. Next, the positions of the corresponding orthologs in stickleback were compared with all the positions of the BLASTZ hits. If one hit is less that 100 kb away from the identified orthologs and no more than five genes are located in between, the hit is retained. To remove false positives due to un-annotated genes, if more than one hit per gene is found within a window of 300 kb, all the hits are discarded.

#### GO filtering

We define developmental genes as genes with the following GO annotation: GO:0045165 (cell fate commitment), GO:0032502 (developmental process), GO:0030528 (transcription regulator activity), and GO:0003700 (transcription factor activity) as well as the descendant annotations as defined by the Open Biomedical Ontologies (version 1.2) [50].

#### Assessment of reading frames

The nucleotide regions on the stickleback genome corresponding to the BLASTZ hits were aligned to the corresponding human exon using BLASTx. If the resulting alignment span of the entire stickleback region within one frame and no stop codon can be found, the region is discarded.

### Bioinformatic Analysis of the Candidate RR

#### Assessment of reading frames

Using the human exon coordinates (Table S1), we retrieved the 46-way multiz hg19 alignments for mouse (*Mus musculus*), chicken (*Gallus gallus*), and xenopus (*Xenopus tropicalis*). Missing sequences (medaka (*Oryzias latipes*), ciona (*Ciona savignyi*)) were retrieved using EnsEMBL v.49, and the orthologous sequences from elephant shark (*Callorhinchus milii*) were retrieved using the homepage of the elephant shark genome project (http://esharkgenome.imcb.a-star.edu.sg) [51]. If no orthologous exon was annotated, tBLASTn was used to retrieve the corresponding regions. The sequences were translated in the reading frame corresponding to the human exon, and an alignment of the orthologous AA sequences was performed (CLUSTALW). For *DOCK9* the 5′UTR was removed in all species analysed. The human *TTC29* exon extends over two exons in the ciona lineage; thus the coding sequence of both ciona exons was fused to do the translation. In medaka, no t*tc29* gene could be found.

#### Multiple alignments, percentage identity/similarity, and Ka/Ks

Sequences were retrieved as described above. The sequences missing from the multiz alignments were added subsequently by global alignment (cost matrix 65% similarity (5.0/−4.0), gap open/extension penalty: 12/3). The percentage identity/similarity to the human exon sequence was calculated using the alignments from pairwise BLASTn (for the nucleotide identity, default parameters) and tBLASTx (for the AA similarity, word size parameter set to 2). Percentages were calculated using the alignable length of the human exon as reference. The Ka/Ks ratio [33] was calculated using the alignable length of the human exon as reference sequence. Because the RRs contain elements that disrupt the ORF (see Assessment of Reading Frames), indels and stop codons were removed prior to calculating the Ka/Ks. Calculations were done using the PAML package included in the PAL2NAL tool of the Bork-Group at EMBL (http://www.bork.embl.de/pal2nal/#RunP2N) [52].

#### Ancestral reconstruction and TFBS composition

Using the human exon coordinates (Table S1), we retrieved the 46-way multiz hg19 alignments. Missing sequences were manually added to the alignment as described above. From this alignment, the predicted ancestral sequence at the root of the bony vertebrates was reconstructed using the Prequel package (default parameters) [53]. Next, we searched for TFBSs in the medaka RR sequences using the Jaspar database (restricting to the Jaspar core vertebrata, 80% relative profile score threshold) [54] and kept only those binding sites that are specific to the teleosts and absent from all the other vertebrate sequences, including the predicted ancestral reconstruction.

### Experimental Methods

#### Medaka stocks

Medaka (*Oryzias latipes*) strains CAB and Heino were kept in closed stocks at EMBL Heidelberg and University of Heidelberg as described [55]. In short, fish were maintained in a constant recirculating system at 28°C on a 14 h light/10 h dark cycle. Pairwise mating was performed and collected embryos were kept at room temperature until hatched.

#### Cloning of candidates and enhancer assay

Chromosomal coordinates and species (assembly) of all cloned and tested fragments are listed in Table S3. Genomic candidate regions (extended to a maximum of 200 bp on each side) tested in the enhancer assay were amplified from genomic DNA of medaka, inbred CAB strain (extraction described in [56]), mouse (C57BL/6 strain, kind gift from F. Spitz), and elephant shark (*Callorhinchus milii*, kind gift from B. Venkatesh) using standard PCR methods. For the dock9 mouse and shark exon constructs, only the exon and 200 bp downstream sequence could be cloned. The 200 bp upstream sequence corresponds to a repeat and could not be amplified. The deletion-constructs were generated by applying a PCR-driven “splicing by overlap extension” approach [57]. For the deletion constructs, in all reporter gene constructs the sequence corresponding to the human exon (the putative RR) was spliced and the flanking genomic sequences were fused. Coordinates of the fused fragments are given in Table S3.

The enhancer assay was performed as described in detail in [21]. In short, genomic sequences were cloned into a transgenesis-vector upstream of a zebrafish hsp70 minimal promoter and GFP reporter gene flanked by I-SceI Meganuclease sites using standard cloning techniques [58]. The constructs were sequenced in order to verify the sequence and the orientation of the cloned regions. Deletions and orthologous constructs were cloned in the same orientation relative to the reporter gene compared to the RR constructs. Meganuclease-mediated transgenesis by injection into one-cell stage medaka embryos (heino or cab strains) was performed as described in [59]. The hsp70 core promoter triggers a strong and specific lens expression from stage 28 on [22], and this feature is used to calculate the percentage of specific expression (Table S3). Stable transgenic lines for all positive enhancer constructs were obtained. Images of transient/stable transgenic embryos were taken using an Olympus MVX10 fluorescence microscope with a Leica DC500 camera or a Leica SPE confocal microscope (10× dipping lens). Images were assembled and processed using ImageJ and Adobe Photoshop. All confocal images displayed are Z-projections of stacks.

#### Mouse transgenic enhancer assay

The mouse sequences orthologous to the RR were cloned upstream of the human β-globin minimal promoter-driven LacZ reporter gene [60]. The constructs were sequenced in order to verify the sequence and the orientation of the cloned regions. The sequences were cloned in the same orientation relative to the reporter gene compared to the RR constructs. Chromosomal coordinates of the cloned and tested mouse fragments are listed in Table S3 (column 7). To evaluate what embryonic developmental stage to test for enhancer activity, we analyzed the expression pattern of the predicted target gene and compared those patterns with the enhancer activity of the RRs: For the ttc29 locus, the medaka enhancer is active in the retina and optic rectum. The putative target gene for this enhancer is *Pou4f2*. The mouse *Pou4f2* is expressed in the hindbrain and retina from E10.5 to after birth [61]. We therefore assayed at embryonic stage E12.5. For the dock9 locus, the medaka enhancer is active in the cerebellum. The putative target genes for this enhancer are *Zic2* and *Zic5*. The mouse *Zic2* and *Zic*5 are expressed in the hindbrain from stage E10.5 to after birth [61]. We therefore looked at embryonic stage E12.5. For the ccdc46^RR^, the medaka enhancer is active in the forebrain. The putative target gene for this enhancer is *axin2*. The mouse *Axin2* is expressed in the telencephalon at stage 14.5 [61]. We therefore looked at embryonic stage E14.5.

Generation of transgenic mice and embryo staining were carried out by Cyagen (Cyagen Bioscience Inc.). The dock9^-exon-mouse^ construct resulted in eight transgenic embryos with two lacZ positive embryos in inconsistent embryonic domains. The ccdc46^-exon-mouse^ construct resulted in 11 transgenic embryos with only one lacZ positive embryo. The ttc29^-exon-mouse^ construct resulted in six transgenic embryos with no lacZ positive embryo.

#### Whole-mount in situ hybridization and double-fluorescent whole-mount in situ hybridization

Whole mount in situ hybridization using digoxigenin labelled antisense RNA probes followed by NBT/BCIP colour detection was performed as previously described [62]. Template cDNA clones were obtained from the medaka full-length cDNA expression library of the Wittbrodt group [62]. The following clones were used to generate the labelled riboprobes: FOE002-P00099-DPE-F_B12 (*pou4f2*, genomic location chr1:22399713-22401052), FOE002-P00076-DPE-F_B12 (*zic2*, genomic location chr21:9245812-9248089), FOE002-P00108-DPE-F_N08 (*zic5*, genomic location chr21:9252145-9254638), and FOE002-P00056-DPE-F_H05 (*axin2 (1 of 2)*, genomic location chr1:4554420-4565844). For genes without a clone in the library, template fragments for in vitro transcription were directly amplified from generated cDNA and cloned into a pTOPO vector (Invitrogen). Total RNA was extracted from 5-d-old embryos using TRIZOL (Invitrogen), and reverse transcription was performed using the Superscript III enzyme (Invitrogen). The following primers were used to amplify cDNA fragments of the genes *ednra (1 of 2)* (fwd: TACAGGGCTGTAGCATCTTGGAGCAG, rev: CGTGTTGACGTTGTTGGGTTCTGG), *clybl* (fwd: GGTAGAAGAGCTCGCAGATGTCTATATG, rev: CTGGCGCAGAAGTCGTCTGAGCC), and *frmpd1* (fwd: ACAGAGAATCCACTCTCCACGTCTACG, rev: TTGGATTTTGTGCTCTGCAGGGATG). In vitro transcription to generate antisense riboprobes was performed using sp6, T3, and T7 RNA polymerases (Roche). Images of whole-mount in situ hybridizations were taken using a Zeiss Axiophot Microscope with a Leica DC500 camera.

Double fluorescent in situ hybridization using digoxygenin-labelled probes against the candidate gene (see above) and a fluorescein-labelled antisense RNA probe generated against GFP were performed as described in [62]. The probes were visualized using Fast Red staining (Roche) and the TSA-Kit (PerkinElmer) as in [62].

Imaging of double-fluorescent whole-mount in situ hybridizations was done using a Leica SPE confocal mircroscope with a 10× dipping lens. Images were assembled and processed using ImageJ and Adobe Photoshop. All confocal images displayed are Z-projections of stacks. Brightness and contrast were adjusted uniformly across the entire image.

## Supporting Information

Figure S1 Overview of the algorithm to identify RR candidate regions. Filtering steps to accurately identify scenarios leading to the appearance of an RR as described in Figure 1B. Homologous regions of all human coding exons were located on the stickleback repeat-masked, non-exonic genome using BLASTZ. Further filtering steps were performed in order to only select putative RRs. Number of remaining hits after each filtering step is shown on the right. See Materials and Methods, Computational Pipeline for more details. The coordinates of the stickleback blastz hits and the corresponding medaka RR candidates are listed in Table S1.
(PDF)

Figure S2 The enhancer activity of the RRs in juvenile medaka fish. In all four cases, the enhancer activity of the RR is maintained in the fish after hatching with similar expression domains as in the embryo. (A) ttc29^RR^ shows activity in the optic tectum (ot) and retina (re), (B) dock9^RR^ shows activity in a part of the cerebellum (ce), (C) ccdc46^RR^ shows activity in the fore- and hindbrain, and (D) fam44b^RR^ shows activity in multiple structures in the brain. The lens expression in all larvae is attributed to the activity of the hsp70 minimal promoter (see Materials and Methods) All larvae are shown in dorsal view, anterior is oriented to the left. Yellow patches correspond to the natural chromatophores in medaka fish.
(PDF)

Figure S3 Assessment of the enhancer activity of the deletion constructs. (A–D) Genomic coordinates of the medaka RR enhancer constructs (green bars) and the deletion constructs, in which the RR corresponding to the length of the human exon was removed (blue bars). Expression patterns of the RR enhancer constructs are shown in Figure 2. (E–H) Deletion of the RR from the ttc29^RR^ and dock9^RR^ constructs lead to a loss of enhancer activity (E, F), while deletion of the RR from the ccdc46^RR^ construct shows a severely altered expression pattern (G). Deletion of the RR from the fam44b^RR^ construct shows a similar expression as the full fam44b^RR^ construct (H). The lens expression is attributed to the activity of the hsp70 minimal promoter (see Materials and Methods). All medaka embryos are shown in dorsal view, anterior is oriented to the left (stages 29 to 32).
(PDF)

Figure S4 Comparison of the expression of the RR-driven GFP reporter lines and the in situ expression patterns of the flanking genes in medaka. (A, C, G) GFP expression driven by the RR in stable transgenic embryos, stage 32 (A,C) and stage 29 (G). The lens expression is attributed to the activity of the hsp70 minimal promoter (see Material and Methods). (B, D, E, F, H) Whole mount in situ hybridizations performed on wild-type embryos with a DIG-labelled antisense RNA probe directed against the genes flanking the RRs. GFP driven by the ttc29^RR^ and the flanking gene *pou4f2* show expression in the optic tectum (A, B), while dock9^RR^-driven GFP and the flanking genes *zic2* and *zic5* are expressed in the cerebellum (C, D, E). The reporter gene under control of ccdc46^RR^ shows expression in the forebrain, as well as *axin2 (1 of 2)*, the gene flanking the ccdc46^RR^ (G, H). *frmpd1* does not show an overlap with ccdc46^RR^-driven GFP expression (F, G). All medaka embryos are shown in dorsal view, anterior is oriented to the left.
(PDF)

Figure S5 Assessment of the reading frames and AA alignments of sequences orthologous to the RR candidates. (A) Assessment of the reading frame in various species: The RRs and the orthologous regions of the RRs in various species are retrieved and the frame equivalent to the human coding frame is scanned for stop codon(s). As expected (see filtering procedure), the reading frame of all medaka RRs is disrupted (magenta squares), while an open reading frame (ORF) is present in all the other species (green squares). Importantly, an ORF is found in at least one outgroup species of the teleost/tetrapod lineage (elephant shark or ciona) for all candidate RRs. The numbers above the squares indicate the reading frame of the human exon. No homologous coding sequence for ttc29^RR^ could be found in medaka and shark. (B) Amino-acid (AA) alignments of the coding exons corresponding to the RRs in various species including one out-group species of the teleost/tetrapod lineage (see Materials and Methods, Bioinformatic Analysis of the Candidate RRs).
(PDF)

Figure S6 Alignments of the medaka RRs to the orthologous human exon (as reference) and other species and assessment of selective pressures. The alignments spanning the human exon coordinates were done by retrieving the 46-way multiz hg19 alignments for selected species. The medaka RRs, medaka coding exon, and the coding exon of an outgroup species were added to the initial alignment subsequently (see Materials and Methods, Bioinformatic Analysis of the Candidate RRs). In the case of dock9^RR^ the alignment shown spans the length of the shark coding part of the exon (shortest coding part). Percentage identity at the nucleotide (NT) level (blue) and the percentage identity/similarity at the AA level (red) were calculated between human and the other sequences. The medaka RRs show a higher identity on the NT level than on the AA level (red arrow), contrasting with regions in other species, where the selective pressure is acting on the AA level. The Ka/Ks ratio between human and the medaka RRs, the medaka coding exon, and the coding exon of an outgroup species (ciona for *TTC29* or shark for *DOCK9 and CCDC46*) were calculated. Higher synonymous amino-acid substitution rates were found at the coding loci (medaka exon and outgroup species) compared to the RR loci, indicating the non-coding nature of the RR candidates.
(PDF)

Figure S7 The number of putative transcription factor binding sites (TFBSs) is lower in coding exons compared to non-exonic regions and experimentally validated enhancers. (A) For most structural classes of transcription factors, a higher number of putative binding sites is found in medaka non-coding regions (regions randomly picked and regions directly flanking the coding exon) than in coding exons, suggesting that coding sequences are less likely to acquire enhancer function compared to non-coding sequences. The total number of binding sites found is 21,480 for the coding exon dataset, 38,840 for the random dataset, and 47,203 for the exon-flanking dataset. (B) The number of binding sites per 200 bp (S200) tend to be lower for exons compared to experimentally validated enhancers. Both distributions show an overlap of only 60% (regulatory potential of exons: 60%). The S200 of the ccdc46^RR^, ttc29^RR^, and dock9^RR^ are also represented. (C) Conversely, the distribution of S200 of non-coding sequences is very similar to the enhancers reaching the regulatory potential of non-coding regions is 95% (C) (see Text S1 for more details).
(PDF)

Figure S8 Predicted TFBSs specific to the teleost RR sequences. Alignment of the medaka RRs to other teleosts and annotation of the predicted TFBSs that are specific to the teleost sequences and absent from all the other vertebrate sequences, including the predicted ancestral reconstructions.
(PDF)

Figure S9 A mouse conserved p300-bound region flanking the *Ccdc46* exon and its orthologs drive reporter gene expression in the medaka forebrain. The mouse p300 sequence (light green bar, upper panel) encompassing the predicted p300-bound enhancer (orange bar, upper panel [38]) and Ccdc46 exonic sequence (orthologous to the ccdc46^RR^) drives GFP expression in the forebrain and a domain in the hindbrain (A). The expression pattern remains unchanged (B) when deleting the exonic sequence from the construct (blue bar, upper panel). The *Ccdc46* exon alone (dark green bar, upper panel) does not show enhancer activity (Figure 4B). The elephant shark sequence orthologous to the mouse p300-bound sequence shows a similar expression pattern as the mouse p300 sequence (C). A construct containing the medaka orthologous sequence of the mouse p300-bound region (orange bar, lower panel) also shows enhancer activity in the forebrain and parts of the optic tectum, hindbrain, and rhombic lips (D). For clarity we included the coordinates of the ccdc46^RR^ and the ccdc46^RR^ delta RR constructs (dark green and blue bars, lower panel) previously assayed (Figure 2C and Figure S3C,G). The genomic coordinates of the tested constructs are given in Table S3.
(PDF)

Table S1 List of four putative RRs. The human gene ID corresponds to the gene lost and replaced by a predicted RR in fish. The genomic coordinates correspond to the human exon (GRCh37), the stickleback (BROAD S1) BLASTZ hit of the computational pipeline, and the experimentally validated medaka RR (MEDAKA1). To calculate the pairwise identity, the number of identical nucleotides was divided by the length of the shortest sequence.
(PDF)

Table S2 List of the genes kept in synteny between human and fish and ciona EST IDs. The human gene ID corresponds to the gene lost and replaced by the predicted RR in fish. The syntenic genes are genes which are kept in synteny between human and fish; in bold are those genes with a developmental GO annotation (see Material and Methods). The EST IDs in *ciona intestinalis* are listed in the last column.
(PDF)

Table S3 Injection statistics of the reporter constructs. For each construct (column 1) we recorded the number of injected embryos (column 2), the number of embryos showing GFP expression in the lens indicative of the successful genomic integration of the construct (column 3), and the number of embryos showing GFP expression specific to the enhancer (expression outside the lens, column 4). From the values of column 3 and 4 the percentage of specific expression due to the activity of the enhancer is calculated (column 5). Columns 6 and 7 indicate the genomic coordinates of the tested regions and the corresponding assembly, respectively. The enhancer constructs include the extended RR region, while in the delta RR constructs the region corresponding to the human exon was deleted (Materials and Methods and Figure S3). The p300 constructs are described in Figure S9 and the main text.
(PDF)

Text S1 Estimation of the number of de novo enhancers across the genome.
(DOC)

## Abbreviations

AA: amino acids
GFP: green fluorescent protein
MYA: million years ago
RR: recycled region
TFBS: transcription factor binding sites
WGD: whole genome duplication
