# Supplementary figures and images for: De Novo Genesis of Enhancers in Vertebrates
Source: PLoS Biol. 2011 Nov 1;9(11):e1001188. doi: 10.1371/journal.pbio.1001188 (PMC3206014; doi:10.1371/journal.pbio.1001188)

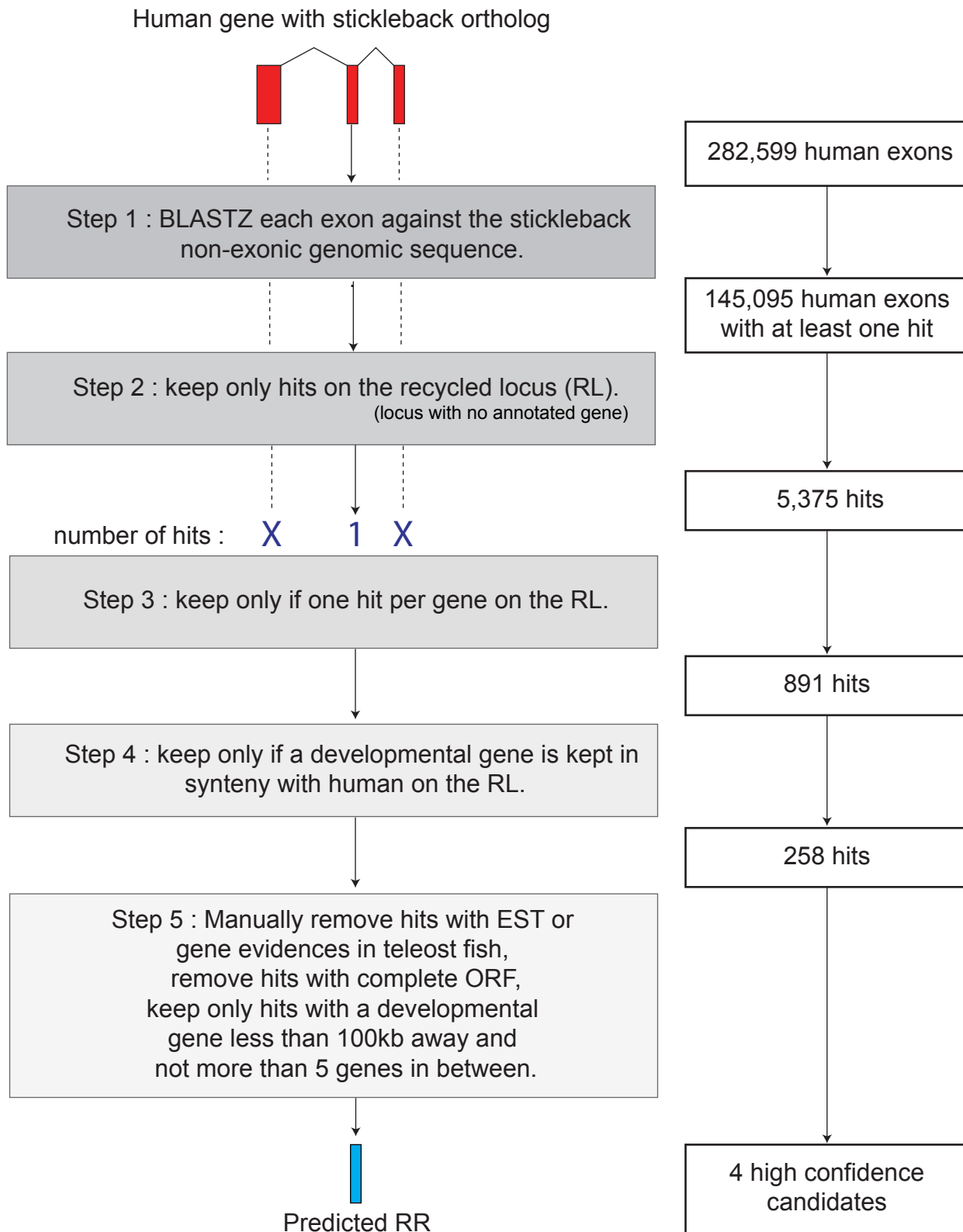

Supplement: Figure S1 — Overview of the algorithm to identify RR candidate regions. Filtering steps to accurately identify scenarios leading to the appearance of an RR as described in Figure 1B. Homologous regions of all human coding exons were located on the stickleback repeat-masked, non-exonic genome using BLASTZ. Further filtering steps were performed in order to only select putative RRs. Number of remaining hits after each filtering step is shown on the right. See Materials and Methods, Computational Pipeline for more details. The coordinates of the stickleback blastz hits and the corresponding medaka RR candidates are listed in Table S1. (PDF) [file pbio.1001188.s001.pdf]

**Figure S2** Eichenlaub and Ettwiller 2011

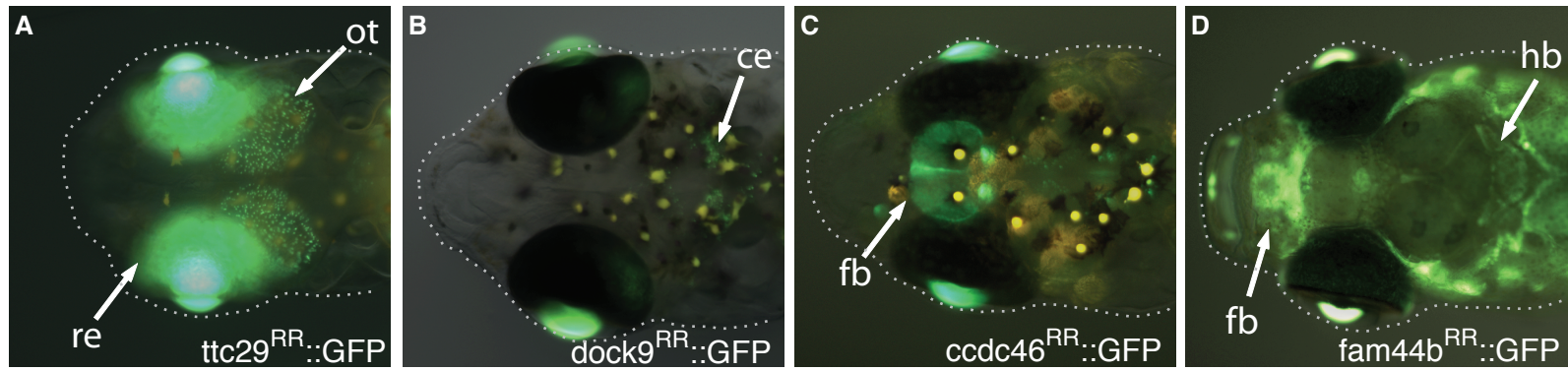

Supplement: Figure S2 — The enhancer activity of the RRs in juvenile medaka fish. In all four cases, the enhancer activity of the RR is maintained in the fish after hatching with similar expression domains as in the embryo. (A) ttc29RR shows activity in the optic tectum (ot) and retina (re), (B) dock9RR shows activity in a part of the cerebellum (ce), (C) ccdc46RR shows activity in the fore- and hindbrain, and (D) fam44bRR shows activity in multiple structures in the brain. The lens expression in all larvae is attributed to the activity of the hsp70 minimal promoter (see Materials and Methods) All larvae are shown in dorsal view, anterior is oriented to the left. Yellow patches correspond to the natural chromatophores in medaka fish. (PDF) [file pbio.1001188.s002.pdf]

**Figure S4** Eichenlaub and Ettwiller 2011

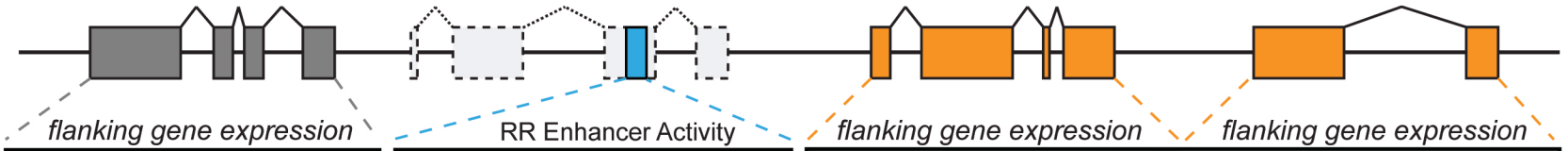

no expression

*ednra* (1 of 2)

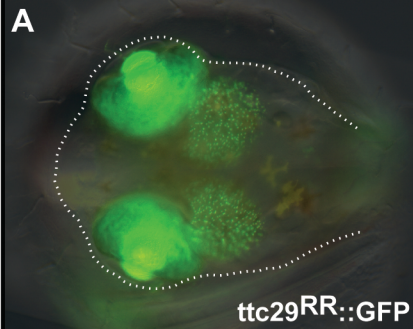

no expression

*clybl*

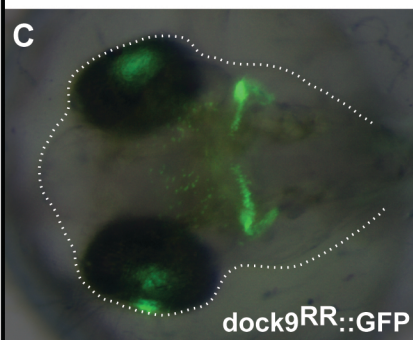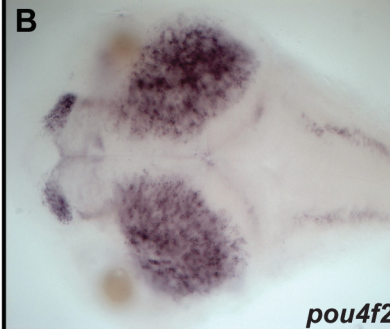

n.d.

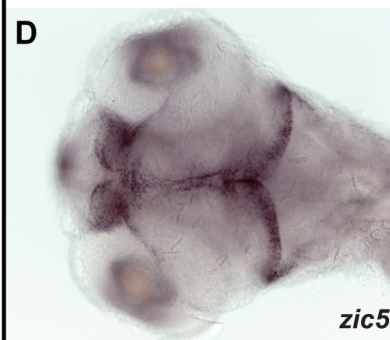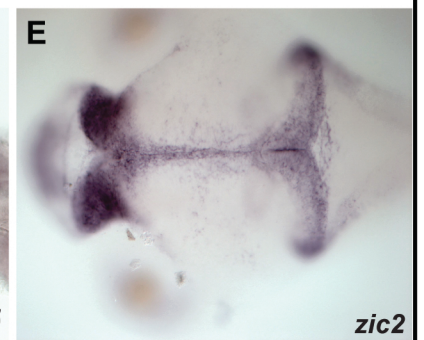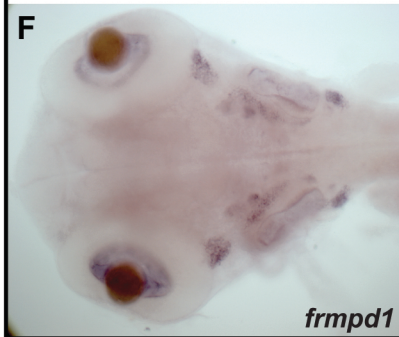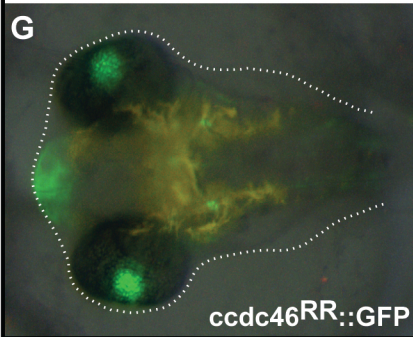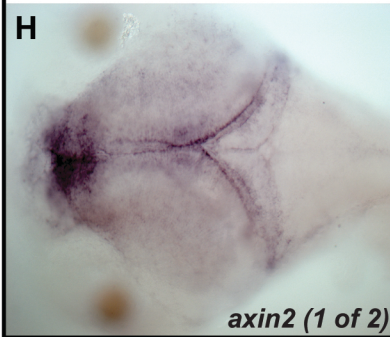

n.d.

Supplement: Figure S4 — Comparison of the expression of the RR-driven GFP reporter lines and the in situ expression patterns of the flanking genes in medaka. (A, C, G) GFP expression driven by the RR in stable transgenic embryos, stage 32 (A,C) and stage 29 (G). The lens expression is attributed to the activity of the hsp70 minimal promoter (see Material and Methods). (B, D, E, F, H) Whole mount in situ hybridizations performed on wild-type embryos with a DIG-labelled antisense RNA probe directed against the genes flanking the RRs. GFP driven by the ttc29RR and the flanking gene pou4f2 show expression in the optic tectum (A, B), while dock9RR-driven GFP and the flanking genes zic2 and zic5 are expressed in the cerebellum (C, D, E). The reporter gene under control of ccdc46RR shows expression in the forebrain, as well as axin2 (1 of 2), the gene flanking the ccdc46RR (G, H). frmpd1 does not show an overlap with ccdc46RR-driven GFP expression (F, G). All medaka embryos are shown in dorsal view, anterior is oriented to the left. (PDF) [file pbio.1001188.s004.pdf]

Figure S8 Eichenlaub and Ettwiller 2011

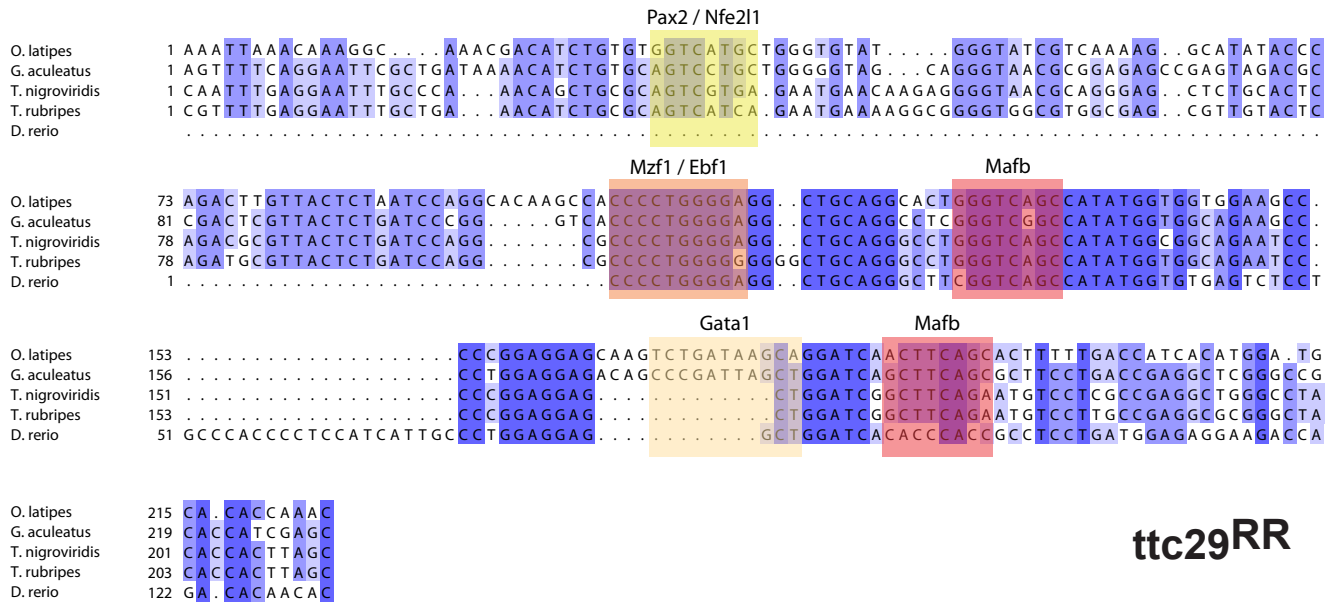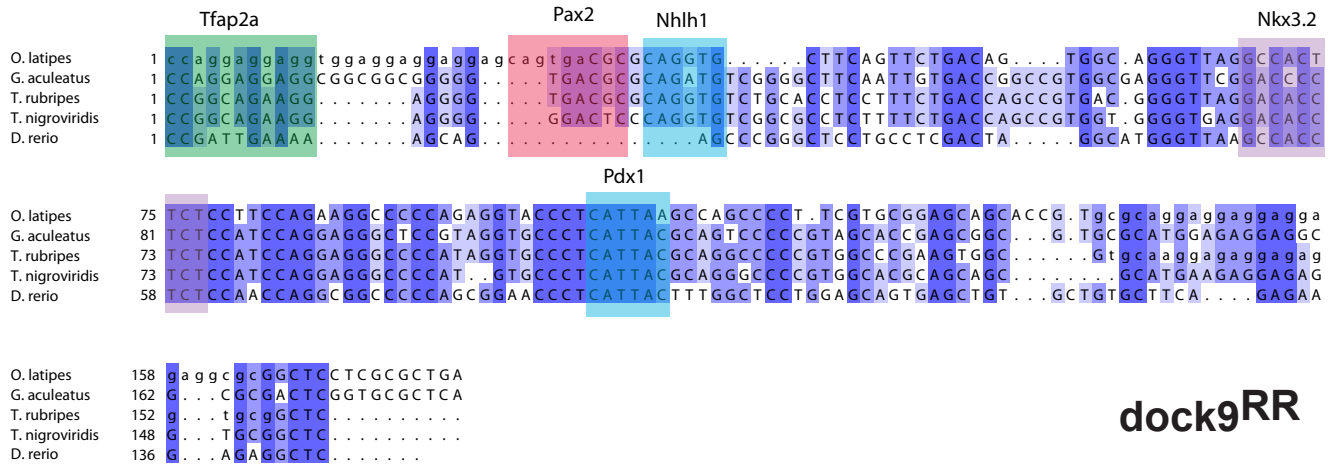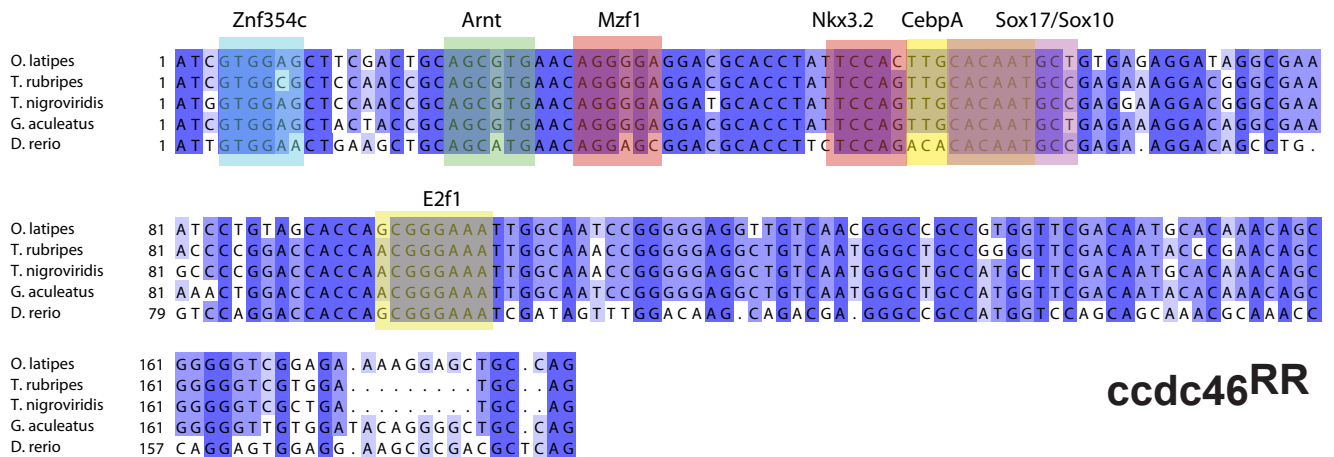

Supplement: Figure S8 — Predicted TFBSs specific to the teleost RR sequences. Alignment of the medaka RRs to other teleosts and annotation of the predicted TFBSs that are specific to the teleost sequences and absent from all the other vertebrate sequences, including the predicted ancestral reconstructions. (PDF) [file pbio.1001188.s008.pdf]
