# Supplementary material for: De Novo Genesis of Enhancers in Vertebrates
Source: PLoS Biol. 2011 Nov 1;9(11):e1001188. doi: 10.1371/journal.pbio.1001188 (PMC3206014; doi:10.1371/journal.pbio.1001188)
Supplement: Figure S3 — Assessment of the enhancer activity of the deletion constructs. (A–D) Genomic coordinates of the medaka RR enhancer constructs (green bars) and the deletion constructs, in which the RR corresponding to the length of the human exon was removed (blue bars). Expression patterns of the RR enhancer constructs are shown in Figure 2. (E–H) Deletion of the RR from the ttc29RR and dock9RR constructs lead to a loss of enhancer activity (E, F), while deletion of the RR from the ccdc46RR construct shows a severely altered expression pattern (G). Deletion of the RR from the fam44bRR construct shows a similar expression as the full fam44bRR construct (H). The lens expression is attributed to the activity of the hsp70 minimal promoter (see Materials and Methods). All medaka embryos are shown in dorsal view, anterior is oriented to the left (stages 29 to 32). (PDF) [file pbio.1001188.s003.pdf]

A

Figure S3 Eichenlaub and Ettwiller 2011

Medaka ttc29^{RR}

chr1:22,443,321 - 22,443,901 (581 bp) / deleted region : chr1:22,443,497 - 22,443,726 (230 bp)

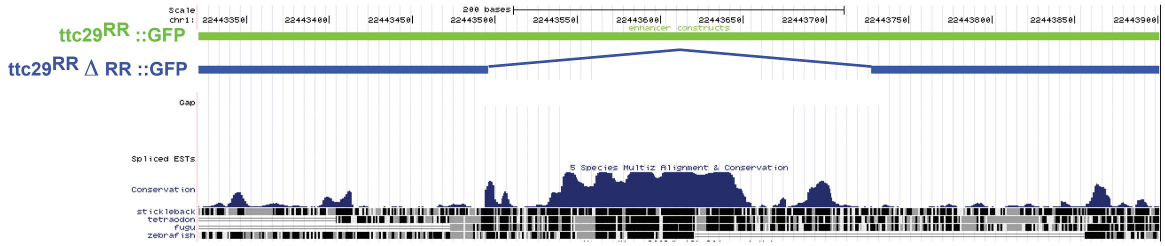

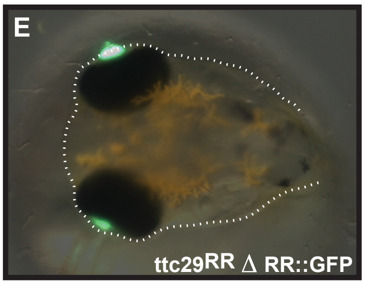

B

Medaka dock9^{RR}

chr21:9,259,960 - 9,260,528 (569 bp) / deleted region: chr21:9,260,151 - 9,260,338 (188 bp)

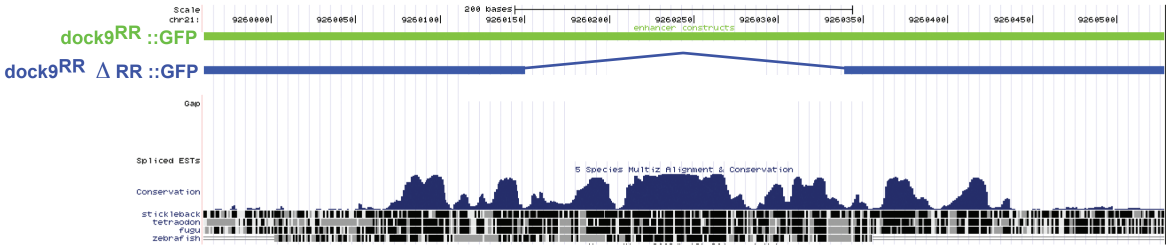

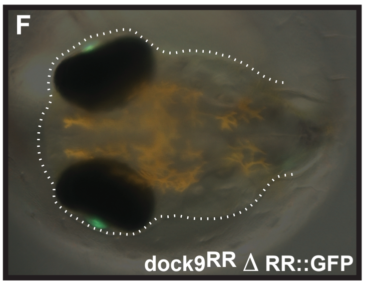

C

Medaka ccdc46^{RR}

chr1: 4,649,989 - 4,650,511 (523 bp) / deleted region: chr1:4,650,131 - 4,650,317 (187 bp)

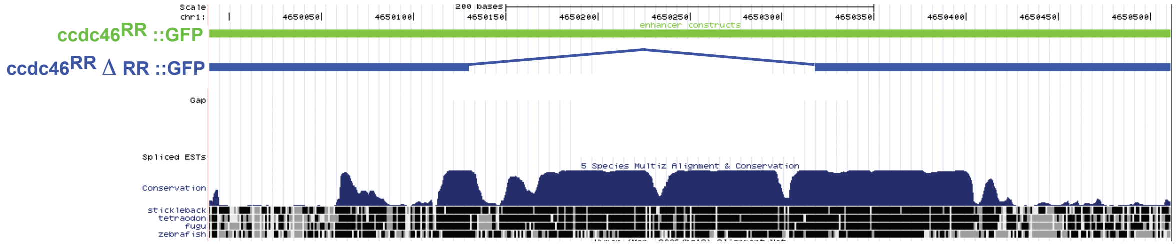

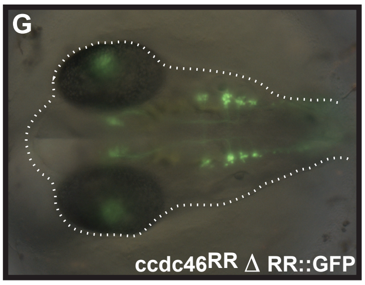

D

Medaka fam44b^{RR}

chr10:16,717,848 - 16,718,337 (490 bp) / deleted region: chr10:16,717,951 - 16,718,149 (199 bp)

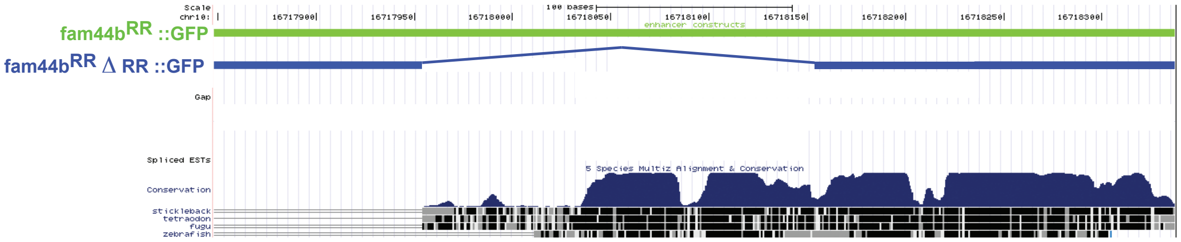

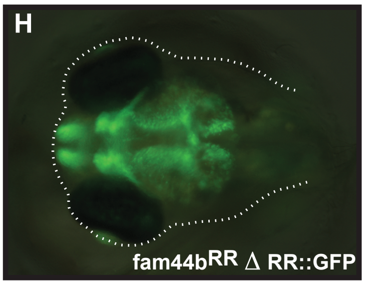

GREEN: RR enhancer construct
BLUE: RR enhancer Δ RR construct

Genome Coordinates: *Oryzias latipes* (HdrR), Oct. 2005 (NIG/UT MEDAKA1/oryLat2)
Conservation tracks: UCSC Genome Browser (http://genome.ucsc.edu/)
