# Supplementary material for: De Novo Genesis of Enhancers in Vertebrates
Source: PLoS Biol. 2011 Nov 1;9(11):e1001188. doi: 10.1371/journal.pbio.1001188 (PMC3206014; doi:10.1371/journal.pbio.1001188)
Supplement: Figure S5 — Assessment of the reading frames and AA alignments of sequences orthologous to the RR candidates. (A) Assessment of the reading frame in various species: The RRs and the orthologous regions of the RRs in various species are retrieved and the frame equivalent to the human coding frame is scanned for stop codon(s). As expected (see filtering procedure), the reading frame of all medaka RRs is disrupted (magenta squares), while an open reading frame (ORF) is present in all the other species (green squares). Importantly, an ORF is found in at least one outgroup species of the teleost/tetrapod lineage (elephant shark or ciona) for all candidate RRs. The numbers above the squares indicate the reading frame of the human exon. No homologous coding sequence for ttc29RR could be found in medaka and shark. (B) Amino-acid (AA) alignments of the coding exons corresponding to the RRs in various species including one out-group species of the teleost/tetrapod lineage (see Materials and Methods, Bioinformatic Analysis of the Candidate RRs). (PDF) [file pbio.1001188.s005.pdf]

**Figure S5** Eichenlaub and Ettwiller 2011

**A**

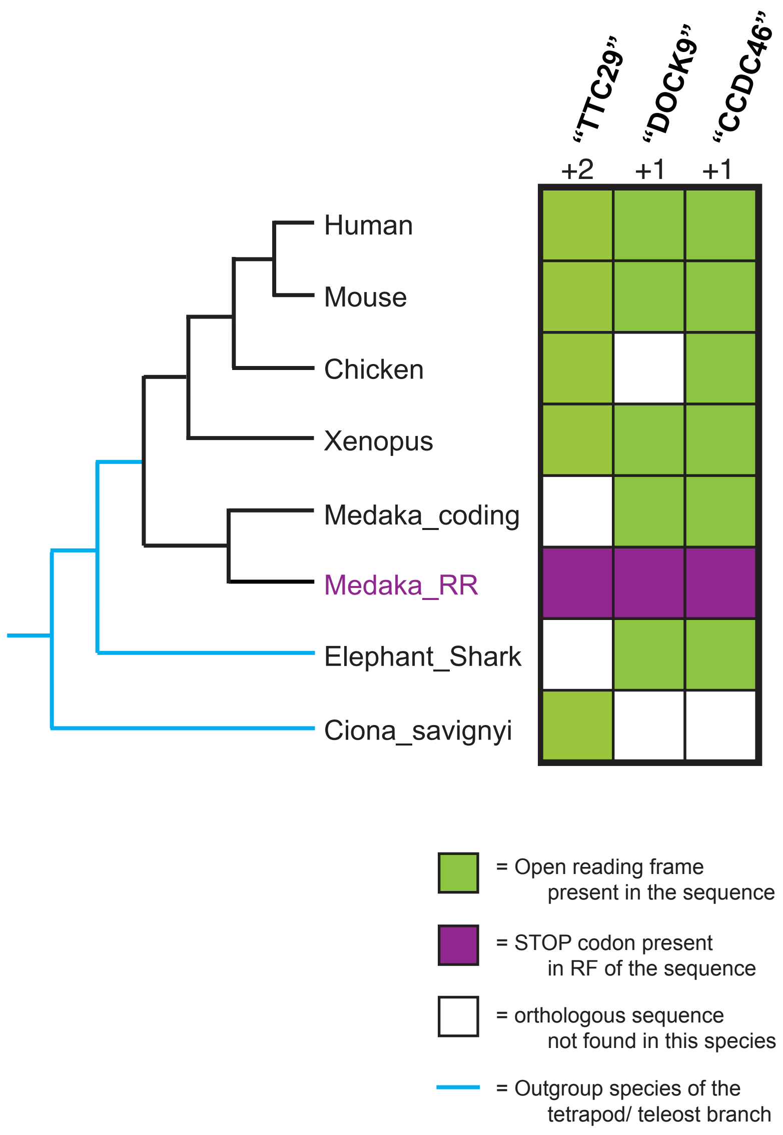


**B**

**TTC29**

```
Human            1 YRNSYKKNICVDMLRDGYHKSFTELFALMERWDALREAARVRS 43
Mouse            1 HRNSYKKSICVDMLRDGFHKSFTELFALMEQWDKLREAAKAQS 43
Chicken          1 NRKSYKENICIDMLRQGYHKSFSELFTLIQKWNALREAAGPGS 43
Xenopus          1 YRNSLKNNICIEMLRKGYHKSFSEVYTVIQRWDALRDAAGPGS 43
Ciona_sav_2exons 1 YRNSYKHNMCVEMLKEGFHKSFDELFNLIEQRKKTRLDAGMDS 43

Human            44 LFWLQKPLEEQPDKLDYLYHYLTRAEDAERK            74
Mouse            44 LFWQQRPLEDQPDKLDNFYHYLTRAEAAERK            74
Chicken          44 AIWQRQSLEEQPDKLDQLYHFLTRAEAAQRA            74
Xenopus          44 AIWHQKPLEEQQEKLDQLQHFLTRAEAAQRA            74
Ciona_sav_2exons 44 VLWHEKPLQEQTEKLDQLWLHLTKAEAALRV            74
```

**DOCK9**

```
Human          1 MSQPPLLPASAETRKFTRALSKPGTAAELRQSVSEVVRGSVLL 43
Mouse          1  MAQQQLPVSTETRKFTRALSKPGTAAELRQSVSEVVRGSVLL 42
Xenopus        1   MSSHHAVHPETRKFTRALSKPGTAAELRQSVSEVVRGSVLM 41
Medaka_coding1 1    MMATVAQPETRKFTRVLSKPGTAAELRQSVSEVVRTSVLV 40
Medaka_coding2 1      MAAAAPEPRKFTRGLNKPGTAAELRQSVSEAVRTSVLM 38
Elephant_Shark 1          MSEVRKFTKRLSKLGTAAELRQSASEAVRNSLFV 34
```

**CCDC46**

```
Human          1 IVELKLEHEQEKTHLLQQHNAEKDSLVRDHEREIENLEKQLRA 43
Mouse          1 IVELKLEHEQEKTHLLQQHSAEKDSLVRDHDREIENLEKQLRA 43
Chicken        1 IVELKLEHEQEKTHLFQQHNAEKDCLVRDHEREIEKLEKQSRA 43
Xenopus        1 MVELRLGHEQERTHLFQQHNNEKESIIKEHEKEIDRLEKQLRS 43
Medaka_coding  1 MAEQRSQHEQERTRLQQQHKAEKDTMVQEHQREVSGVESQARA 43
Elephant_Shark 1 IVELKLEREQEKTYLFQQHSAEKDSLVREHEREIDNLEKQLRA 43

Human          44 ANMEHENQIQEFKKRDAQ                         61
Mouse          44 ANMEHENQIQESKKRDAQ                         61
Chicken        44 AMAEHESRTQECRKRDGQ                         61
Xenopus        44 TVSDHEAKTQAWRQRDAQ                         61
Medaka_coding  44 ALQQHQQHSQEWRKRDAQ                         61
Elephant_Shark 44 AIVEHENQTRQLRKQDTQ                         61
```
