# Supplementary material for: De Novo Genesis of Enhancers in Vertebrates
Source: PLoS Biol. 2011 Nov 1;9(11):e1001188. doi: 10.1371/journal.pbio.1001188 (PMC3206014; doi:10.1371/journal.pbio.1001188)

ttc29RR

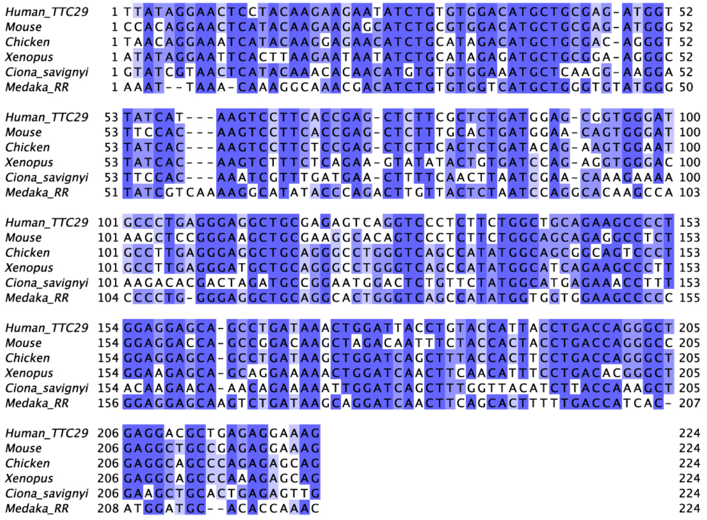

> reference: human exon on chr4:147830178-147830401:-1, GRCh37

% identity / similarity to human exon

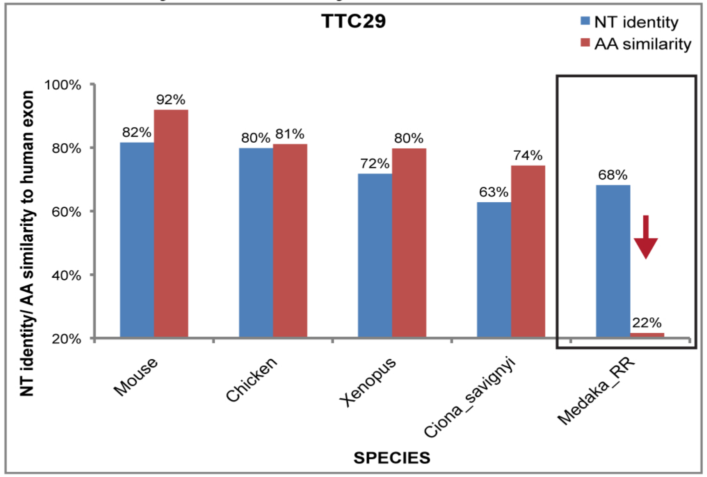

Ka/Ks to human exon

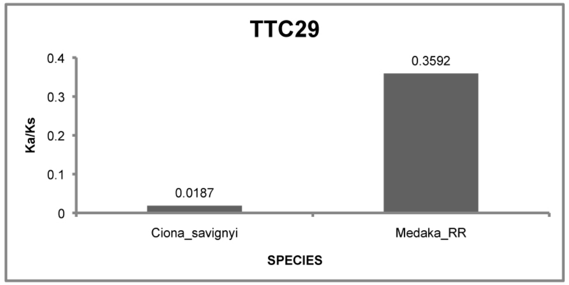

dock9RR

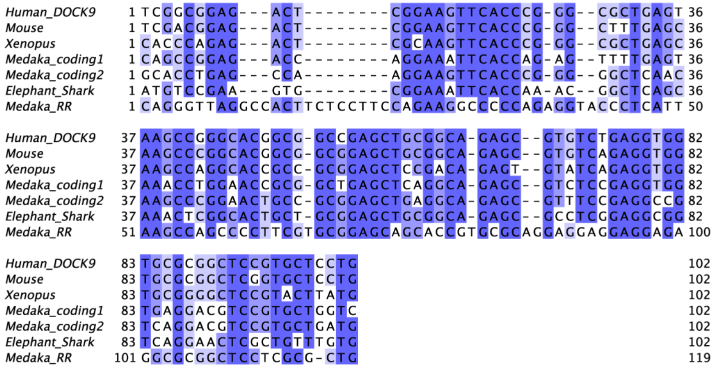

> reference: human exon on chr13:99738477-99738578:-1, GRCh37

% identity / similarity to human exon

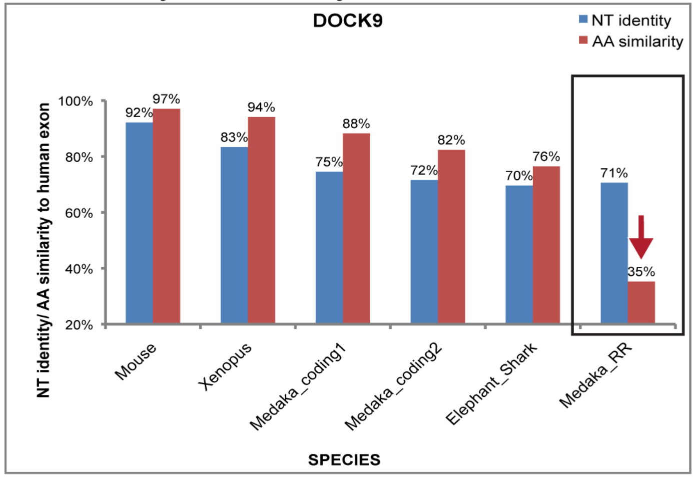

Ka/Ks to human exon

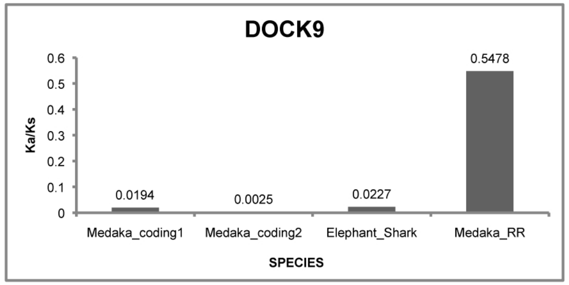

ccdc46RR

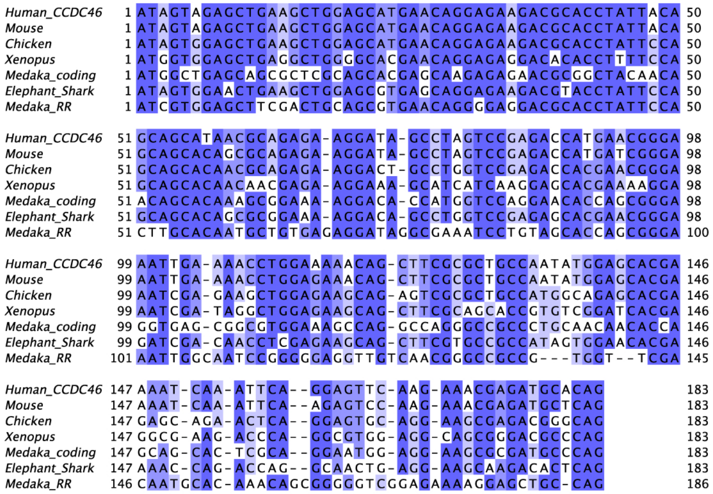

> reference: human exon on chr17:63898270-63898452:-1, GRCh37

% identity / similarity to human exon

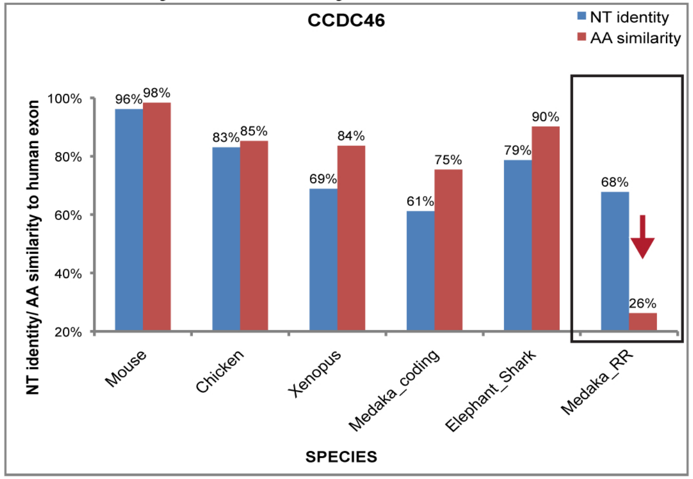

Ka/Ks to human exon

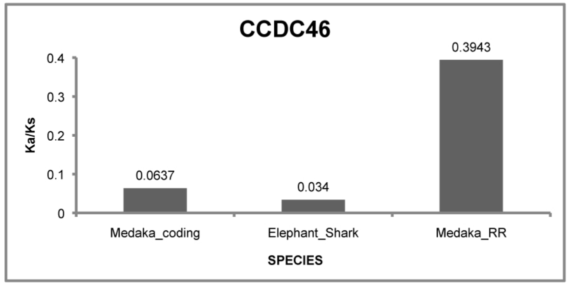

Supplement: Figure S6 — Alignments of the medaka RRs to the orthologous human exon (as reference) and other species and assessment of selective pressures. The alignments spanning the human exon coordinates were done by retrieving the 46-way multiz hg19 alignments for selected species. The medaka RRs, medaka coding exon, and the coding exon of an outgroup species were added to the initial alignment subsequently (see Materials and Methods, Bioinformatic Analysis of the Candidate RRs). In the case of dock9RR the alignment shown spans the length of the shark coding part of the exon (shortest coding part). Percentage identity at the nucleotide (NT) level (blue) and the percentage identity/similarity at the AA level (red) were calculated between human and the other sequences. The medaka RRs show a higher identity on the NT level than on the AA level (red arrow), contrasting with regions in other species, where the selective pressure is acting on the AA level. The Ka/Ks ratio between human and the medaka RRs, the medaka coding exon, and the coding exon of an outgroup species (ciona for TTC29 or shark for DOCK9 and CCDC46) were calculated. Higher synonymous amino-acid substitution rates were found at the coding loci (medaka exon and outgroup species) compared to the RR loci, indicating the non-coding nature of the RR candidates. (PDF) [file pbio.1001188.s006.pdf]
