# Supplementary material for: De Novo Genesis of Enhancers in Vertebrates
Source: PLoS Biol. 2011 Nov 1;9(11):e1001188. doi: 10.1371/journal.pbio.1001188 (PMC3206014; doi:10.1371/journal.pbio.1001188)

Figure S7 Eichenlaub and Ettwiller 2011

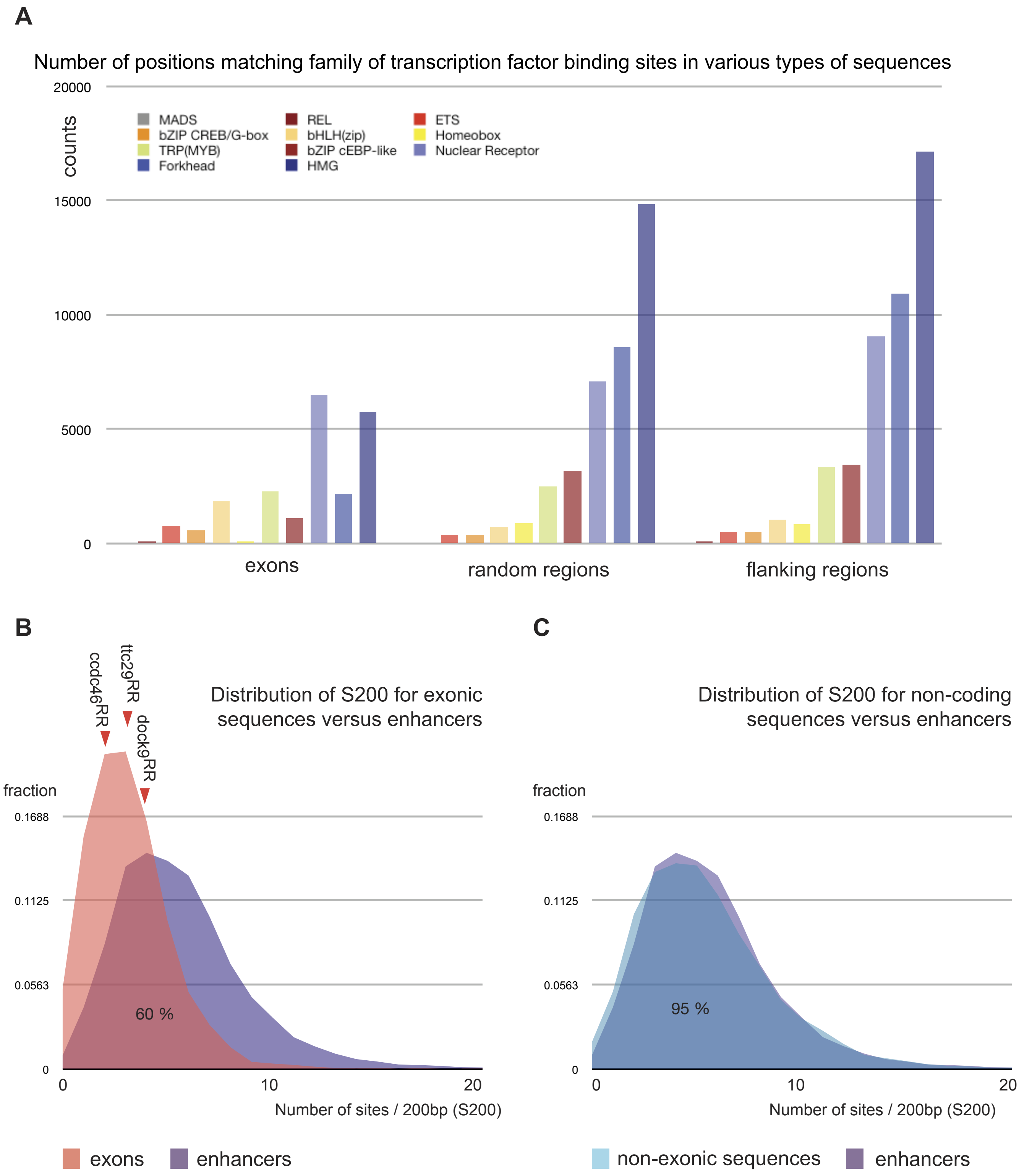

Supplement: Figure S7 — The number of putative transcription factor binding sites (TFBSs) is lower in coding exons compared to non-exonic regions and experimentally validated enhancers. (A) For most structural classes of transcription factors, a higher number of putative binding sites is found in medaka non-coding regions (regions randomly picked and regions directly flanking the coding exon) than in coding exons, suggesting that coding sequences are less likely to acquire enhancer function compared to non-coding sequences. The total number of binding sites found is 21,480 for the coding exon dataset, 38,840 for the random dataset, and 47,203 for the exon-flanking dataset. (B) The number of binding sites per 200 bp (S200) tend to be lower for exons compared to experimentally validated enhancers. Both distributions show an overlap of only 60% (regulatory potential of exons: 60%). The S200 of the ccdc46RR, ttc29RR, and dock9RR are also represented. (C) Conversely, the distribution of S200 of non-coding sequences is very similar to the enhancers reaching the regulatory potential of non-coding regions is 95% (C) (see Text S1 for more details). (PDF) [file pbio.1001188.s007.pdf]
