# Supplementary material for: De Novo Genesis of Enhancers in Vertebrates
Source: PLoS Biol. 2011 Nov 1;9(11):e1001188. doi: 10.1371/journal.pbio.1001188 (PMC3206014; doi:10.1371/journal.pbio.1001188)
Supplement: Figure S9 — A mouse conserved p300-bound region flanking the Ccdc46 exon and its orthologs drive reporter gene expression in the medaka forebrain. The mouse p300 sequence (light green bar, upper panel) encompassing the predicted p300-bound enhancer (orange bar, upper panel [38]) and Ccdc46 exonic sequence (orthologous to the ccdc46RR) drives GFP expression in the forebrain and a domain in the hindbrain (A). The expression pattern remains unchanged (B) when deleting the exonic sequence from the construct (blue bar, upper panel). The Ccdc46 exon alone (dark green bar, upper panel) does not show enhancer activity (Figure 4B). The elephant shark sequence orthologous to the mouse p300-bound sequence shows a similar expression pattern as the mouse p300 sequence (C). A construct containing the medaka orthologous sequence of the mouse p300-bound region (orange bar, lower panel) also shows enhancer activity in the forebrain and parts of the optic tectum, hindbrain, and rhombic lips (D). For clarity we included the coordinates of the ccdc46RR and the ccdc46RR delta RR constructs (dark green and blue bars, lower panel) previously assayed (Figure 2C and Figure S3C,G). The genomic coordinates of the tested constructs are given in Table S3. (PDF) [file pbio.1001188.s009.pdf]

Figure S9 Eichenlaub and Ettwiller 2011

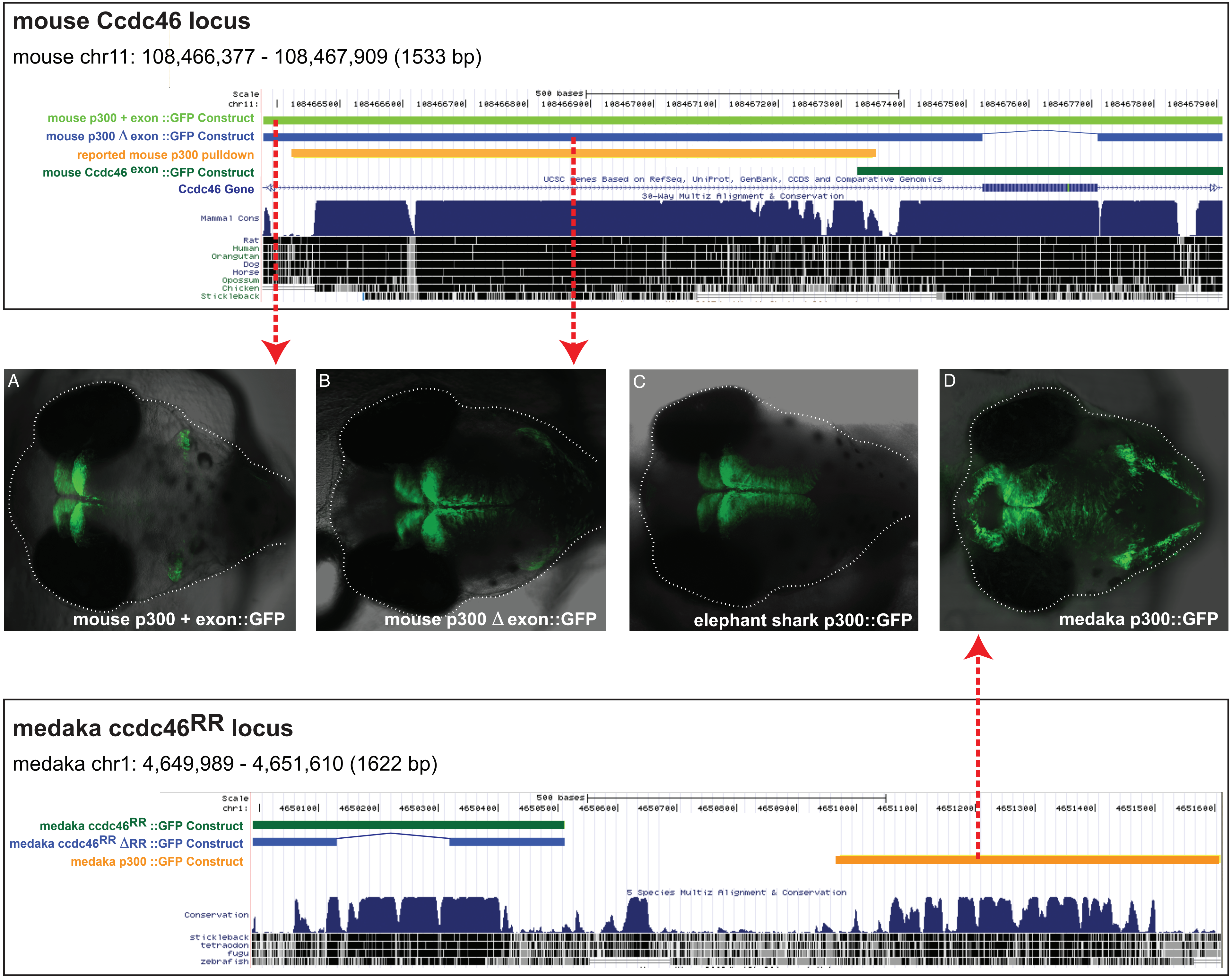

Genome Coordinates
***Oryzias latipes*:**
(NIG/UT MEDAKA1/oryLat2)
Mus musculus:
(NCBI37/mm9)

Conservation tracks:
UCSC Genome Browser
(http://genome.ucsc.edu/)
