# Supplementary material for: De Novo Genesis of Enhancers in Vertebrates
Source: PLoS Biol. 2011 Nov 1;9(11):e1001188. doi: 10.1371/journal.pbio.1001188 (PMC3206014; doi:10.1371/journal.pbio.1001188)
Supplement: Table S1 — List of four putative RRs. The human gene ID corresponds to the gene lost and replaced by a predicted RR in fish. The genomic coordinates correspond to the human exon (GRCh37), the stickleback (BROAD S1) BLASTZ hit of the computational pipeline, and the experimentally validated medaka RR (MEDAKA1). To calculate the pairwise identity, the number of identical nucleotides was divided by the length of the shortest sequence. (PDF) [file pbio.1001188.s010.pdf]

**Candidate RR Region**	**“ttc29^{RR}"**	**“dock9^{RR}"**	**“ccdc46^{RR}"**	**“fam44b^{RR}"**
**Human Gene ENSEMBL ID**	ENSG00000137473 (TTC29)	ENSG00000088387 (DOCK9)	ENSG00000154240 (CCDC46)	ENSG00000145919 (FAM44B)
**Human exon location (GRCh37)**	chr4:147830178-147830401:-1	chr13:99738477-99738660:-1	chr17:63898270-63898452:-1	chr5:173036241-173036437:-1
**Human exon length**	224 bp	184 bp	183 bp	197 bp
**Stickleback BLASTZ hit (BROADS1)**	groupIX:6710625-6710804:1	groupXVI:2860453-2860626:-1	groupIX:12578736-12578860:1	groupIV:13966044-13966125:-1
**Stickleback BLASTZ hit length**	180 bp	174 bp	125 bp	82 bp
**% ID to hs exon (BLASTZ hit)**	70%	66%	72%	73%
**Medaka RR (MEDAKA1)**	chr1:22443500-22443723:-1	chr21:9260151-9260338:1	chr1:4650130-4650315:-1	chr10:16717951-16718149:-1
**Medaka RR length**	224 bp	188 bp	186 bp	199 bp
**% ID to hs exon (Medaka RR)**	68%	69%	68%	60%
