# Supplementary material for: De Novo Genesis of Enhancers in Vertebrates
Source: PLoS Biol. 2011 Nov 1;9(11):e1001188. doi: 10.1371/journal.pbio.1001188 (PMC3206014; doi:10.1371/journal.pbio.1001188)
Supplement: Table S2 — List of the genes kept in synteny between human and fish and ciona EST IDs. The human gene ID corresponds to the gene lost and replaced by the predicted RR in fish. The syntenic genes are genes which are kept in synteny between human and fish; in bold are those genes with a developmental GO annotation (see Material and Methods). The EST IDs in ciona intestinalis are listed in the last column. (PDF) [file pbio.1001188.s011.pdf]

| Candidate RR Region | Human Gene ENSEMBL ID (EntrezGene ID) | Human genes in synteny with stickleback | Ciona EST IDs |
|---|---|---|---|
| **$ttc29^{RR}$** | **TTC29 ENSG00000137473 (83894)** | **POU4F2, EDNRA,** TMEM34, PRMT10, ARHGAP10, LSM6, NR3C2, ZNF827 MMAA, SMAD1, HHIP | **BW280658.1, BW416608, BP024045 (GeneBank)** |
| **$dock9^{RR}$** | **DOCK9 ENSG00000088387 (23348)** | **ZIC2, ZIC5, CLYBL,** EBI2, GPR18, GPR183, TMTC4, NALCN | **---** |
| **$ccdc46^{RR}$** | **CCDC46 ENSG00000154240 (201134)** | **AXIN2,** KPNA2, NOL11, APOH, PRKCA, CACNG5, CACNG4, HELZ, PSMD12, PITPNC1 | **rcijv407d10, rcibd051c09 (Ghost Database)** |
| **$fam44b^{RR}$** | **FAM44B ENSG00000145919 (91272)** | **BNIP1,** ATP6V0E1, RPL26L1, STC2, CPEB4, MSX2, DRD1 | **---** |
