# Supplementary material for: De Novo Genesis of Enhancers in Vertebrates
Source: PLoS Biol. 2011 Nov 1;9(11):e1001188. doi: 10.1371/journal.pbio.1001188 (PMC3206014; doi:10.1371/journal.pbio.1001188)
Supplement: Table S3 — Injection statistics of the reporter constructs. For each construct (column 1) we recorded the number of injected embryos (column 2), the number of embryos showing GFP expression in the lens indicative of the successful genomic integration of the construct (column 3), and the number of embryos showing GFP expression specific to the enhancer (expression outside the lens, column 4). From the values of column 3 and 4 the percentage of specific expression due to the activity of the enhancer is calculated (column 5). Columns 6 and 7 indicate the genomic coordinates of the tested regions and the corresponding assembly, respectively. The enhancer constructs include the extended RR region, while in the delta RR constructs the region corresponding to the human exon was deleted (Materials and Methods and Figure S3). The p300 constructs are described in Figure S9 and the main text. (PDF) [file pbio.1001188.s012.pdf]

Construct	Injected Embryos	Lens Expression (pos. Integration)	Specific Expression Pattern	% of Specific Expression	Genomic Coordinates of Construct	Species (Assembly)
**Medaka ttc29^{RR} ::GFP**	117	40	27	**67.50%**	chr1:22443321-22443901	Medaka (MEDAKA1)
**Medaka ttc29^{RR} ∆ RR ::GFP**	75	17	0	**0.00%**	chr1:22443321-22443496, chr1:22443727-22443901	Medaka (MEDAKA1)
**Mouse Ttc29^{exon} ::GFP (coding ortholog)**	91	34	0	**0.00%**	chr8:80769798-80770621	Mouse (NCBI m37)
**Medaka dock9^{RR} ::GFP**	94	33	17	**51.52%**	chr21:9259960-9260528	Medaka (MEDAKA1)
**Medaka dock9^{RR} ∆ RR ::GFP**	88	26	0	**0.00%**	chr21:9259960-9260150, chr21:9260339-9260528	Medaka (MEDAKA1)
**Medaka dock9^{exon} ::GFP (coding paralog 1of2)**	60	15	0	**0.00%**	chr21:25113257-25113767	Medaka (MEDAKA1)
**Medaka dock9^{exon} ::GFP (coding paralog 2of2)**	78	24	0	**0.00%**	chr2:21810012-21810516	Medaka (MEDAKA1)
**Mouse Dock9^{exon} ::GFP (coding ortholog)**	77	21	0	**0.00%**	chr14:122196512:122196833	Mouse (NCBI m37)
**Elephant Shark dock9^{exon} ::GFP (coding ortholog)**	63	33	0	**0.00%**	contig AAVX01189524.1: 83-300	Elephant Shark (AAVX01000000)
**Medaka ccdc46^{RR} ::GFP**	78	26	16	**61.54%**	chr1:4649989-4650511	Medaka (MEDAKA1)
**Medaka ccdc46^{RR} ∆ RR ::GFP**	160	38	25	**65.79%** (residual activity)	chr1:4649989-4650130, chr1:4650318-4650511	Medaka (MEDAKA1)
**Medaka ccdc46^{exon} ::GFP (coding paralog)**	78	47	0	**0.00%**	chr8:12224568-12225059	Medaka (MEDAKA1)
**Mouse Ccdc46^{exon} ::GFP (coding ortholog)**	84	31	0	**0.00%**	chr11:108467327-108467909	Mouse (NCBI m37)
**Elephant Shark ccdc46^{exon} ::GFP (coding ortholog)**	111	24	0	**0.00%**	contig AAVX01033398.1: 1414-1971	Elephant Shark (AAVX01000000)
**Medaka p300 ::GFP**	68	24	22	**91.67%**	chr1:4650972-4651610	Medaka (MEDAKA1)
**Mouse p300 + exon ::GFP**	82	17	14	**82.35%**	chr11:108466377-108467909	Mouse (NCBI m37)
**Mouse p300 ∆ exon ::GFP**	98	28	22	**78.57%**	chr11:108466377-108467526, chr11:108467710-108467909	Mouse (NCBI m37)
**Elephant Shark p300 ::GFP**	91	23	18	**78.26%**	contig AAVX01033398.1: 1-1606	Elephant Shark (AAVX01000000)
**Medaka fam44b^{RR} ::GFP**	92	35	20	**57.14%**	chr10:16717848-16718337	Medaka (MEDAKA1)
**Medaka fam44b^{RR} ∆ RR ::GFP**	136	28	15	**53.57%**	chr10:16717848-16717950, chr10:16718150-16718337	Medaka (MEDAKA1)

(green box) = enhancer activity overlapping with expression domain of a flanking gene
