# Supplementary material for: De Novo Genesis of Enhancers in Vertebrates
Source: PLoS Biol. 2011 Nov 1;9(11):e1001188. doi: 10.1371/journal.pbio.1001188 (PMC3206014; doi:10.1371/journal.pbio.1001188)
Supplement: Text S1 — Estimation of the number of de novo enhancers across the genome. (DOC) [file pbio.1001188.s013.doc]

**Text S1 Estimation of the number of *de-novo* enhancers across the genome.**

To estimate the rate of *de-novo* genesis of enhancers at a genome scale, we quantified how our methodology restricts -1- the genomic space, -2- the evolutionary time window and -3- the bias inherent to the starting material composed of exons rather than non-exonic sequence:

1. The genomic space: Four percent of the medaka genome is currently composed of coding exons. Considering that 3 out of 4 duplicated genes have lost one copy after WGD [1] around 3% of the genome is derived from lost coding exons which correspond to the starting material used in this study to identify *de-novo* enhancers.
2. The evolutionary time window: In our approach, traceability of the region across evolutionary time is required for the region to be selected. Recycled regions (RRs) are alignable with their coding orthologs only if a sufficiently fast selection for the new regulatory function limits the number of nucleotide changes. To estimate the evolutionary time needed for a neutral region in medaka to loose a significant alignment we use INDELible [2] to simulate sequence evolution with the following parameters : The substitution model has been set to HKY +  with a transition–transversion rate ratio of  2, stationary base frequencies of 0.25 (T), 0.25 (C), 0.25 (A), and 0.25 (G), and continuous gamma rate variation with shape parameter  1. Insertions and deletions have both been set to have an instantaneous rate of 0.1 (relative to an average substitution rate of 1) and the same geometric length distribution with a mean length of 4. The sequence length was set to 179, corresponding to the average exon lengths in medaka [3] and the estimated rate of neutral evolution is set to 0.0146 sites per Myr [1]. For each simulation, we assessed whether a significant alignment can be found using BLASTZ. 100 simulations were performed per 100 000 years increment and the minimum time necessary to loose the majority of the significant alignments was calculated to be 12.7 Mys. Therefore, 12.7 Mys is the average time needed for a neutrally evolving sequence to loose significant alignment with its ancestral sequence.
3. The bias inherent to the starting material: We investigated whether the different motif composition in ancestrally coding sequences affects the potency to acquire enhancer functions. We use the JASPAR FAM database [4] (October 12, 2009 version) consisting of 11 position weight matrices (PWMs) describing shared binding properties of structural classes of transcription factors. The number of positions matching at least 90% for each of the PWM [5] is calculated for (a) 10 000 randomly picked Medaka coding exons from ensembl v49, (b) 10 000 sequences of identical lengths as for the dataset (a) but derived from random locations and (c) 10 000 sequences of identical lengths as for the dataset (a) but directly flanking exons in dataset (a).  The total number of binding sites found is 21480 for dataset (a), 38840 for dataset (b) and 47203 for dataset (c) **(Figure S7A)**.  Taken together this lower number of cryptic TFBSs for most of the structural classes of transcription factors in coding exons compared to non-coding regions suggests that coding sequences are less likely to acquire enhancer function compared to non-coding sequences. To further assess how coding sequences are less likely to acquire enhancer activity, we selected a class of sequences that have been experimentally validated as being enhancers (605 experimentally validated human enhancers from [6] (downloaded 01/15/10)). We make the assumption that the overall density of binding sites in enhancers is conserved from human to fish. For each enhancer, the total number of sites per 200 bp (S200) is calculated. Next, we derived the distribution of S200 across all validated enhancers and set the regulatory potential at 100% if a given dataset shows the same S200 distribution. The distribution of S200 is then calculated for the exonic sequences (dataset a) and flanking sequences (dataset c). The regulatory potential of exons is calculated to be 60% while the regulatory potential of non-exonic regions is calculated to be 95% (**Figure S7 B, C)**. For information, the S200 for ttc29, ccdc46 and dock9 RRs are 2.2, 3.3 and 4.5 sites respectively. We did not find significant differences between duplicated exons and non duplicated exons (data not shown).

Taken together, the three cases of *de-novo* enhancers reported in this study derive from only 3% of the genome over a limited evolutionary time of 12.7 Mys. Extrapolated to the whole genome across 450 Mys of evolution since the tetrapod-teleost split [1], we estimate 3543 *de-novo* enhancers under positive selection. Now further considering that the regulatory potential of exons is only 60%, while non-exonic sequences is 95%, the estimation is now roughly 6,000 (5610) *de-novo* enhancers in teleosts. We note that further studies will be needed to make a more accurate prediction.

***References Text S1***

1. Jaillon O, Aury J-M, Brunet F, Petit J-L, Stange-Thomann N, et al. (2004) Genome duplication in the teleost fish Tetraodon nigroviridis reveals the early vertebrate proto-karyotype. Nature 431: 946-957.

2. Fletcher W, Yang Z (2009) INDELible: a flexible simulator of biological sequence evolution. Mol Biol Evol 26: 1879-1888.

3. Kasahara M, Naruse K, Sasaki S, Nakatani Y, Qu W, et al. (2007) The medaka draft genome and insights into vertebrate genome evolution. Nature 447: 714-719.

4. Sandelin A, Alkema W, Engström P, Wasserman WW, Lenhard B (2004) JASPAR: an open-access database for eukaryotic transcription factor binding profiles. Nucleic Acids Res 32: D91-94.

5. Lenhard B, Wasserman WW (2002) TFBS: Computational framework for transcription factor binding site analysis. Bioinformatics 18: 1135-1136.

6. Visel A, Minovitsky S, Dubchak I, Pennacchio LA (2007) VISTA Enhancer Browser--a database of tissue-specific human enhancers. Nucleic Acids Res 35: D88-92.
